# Mutant TP53 switches therapeutic vulnerability during gastric cancer progression within interleukin-6 family cytokines

**DOI:** 10.1016/j.celrep.2024.114616

**Published:** 2024-08-09

**Authors:** Anne Huber, Amr H. Allam, Christine Dijkstra, Stefan Thiem, Jennifer Huynh, Ashleigh R. Poh, Joshua Konecnik, Saumya P. Jacob, Rita Busuttil, Yang Liao, David Chisanga, Wei Shi, Mariah G. Alorro, Stephen Forrow, Daniele V.F. Tauriello, Eduard Batlle, Alex Boussioutas, David S. Williams, Michael Buchert, Matthias Ernst, Moritz F. Eissmann

**Affiliations:** 1 Olivia Newton-John Cancer Research Institute and School of Cancer Medicine, La Trobe University, Melbourne, VIC 3084, Australia; 2 Central Clinical School, Monash University, Melbourne, VIC 3004, Australia; 3 Department of Gastroenterology, The Alfred Hospital, Melbourne, VIC 3004, Australia; 4 Institute for Research in Biomedicine (IRB Barcelona), The Barcelona Institute of Science and Technology (BIST), 08028 Barcelona, Spain; 5 Centro de Investigación Biomédica en Red de Cáncer (CIBERONC), Barcelona, Spain; 6 Institució Catalana de Recerca i Estudis Avançats (ICREA), Barcelona, Spain; 7 Department of Anatomical Pathology, Austin Health, Heidelberg, VIC 3084, Australia

**Keywords:** TP53, gastric carcinoma, STAT3, cytokine signaling, animal model, organoids, interleukin-6, interleukin-11, metastasis, WNT

## Abstract

Although aberrant activation of the KRAS and PI3K pathway alongside TP53 mutations account for frequent aberrations in human gastric cancers, neither the sequence nor the individual contributions of these mutations have been clarified. Here, we establish an allelic series of mice to afford conditional expression in the glandular epithelium of *Kras*^*G12D*^;*Pik3ca*^*H1047R*^ or *Trp53*^*R172H*^ and/or ablation of *Pten* or *Trp53*. We find that *Kras*^*G12D*^;*Pik3ca*^*H1047R*^ is sufficient to induce adenomas and that lesions progress to carcinoma when also harboring *Pten* deletions. An additional challenge with either *Trp53* loss- or gain-of-function alleles further accelerated tumor progression and triggered metastatic disease. While tumor-intrinsic STAT3 signaling in response to gp130 family cytokines remained as a gatekeeper for all stages of tumor development, metastatic progression required a mutant *Trp53*-induced interleukin (IL)-11 to IL-6 dependency switch. Consistent with the poorer survival of patients with high IL-6 expression, we identify IL-6/STAT3 signaling as a therapeutic vulnerability for TP53-mutant gastric cancer.

## Highlights

• Mutant TP53 licenses progression to invasive stomach carcinoma with synchronous metastasis
• Gain- and loss-of-function TP53 mutants advance gastric cancer upon loss of heterozygosity
• Gastric cancer’s STAT3 addiction switches from IL-11 to IL-6 dependency when being TP53 mutant

## Graphical abstract

**Figure:**
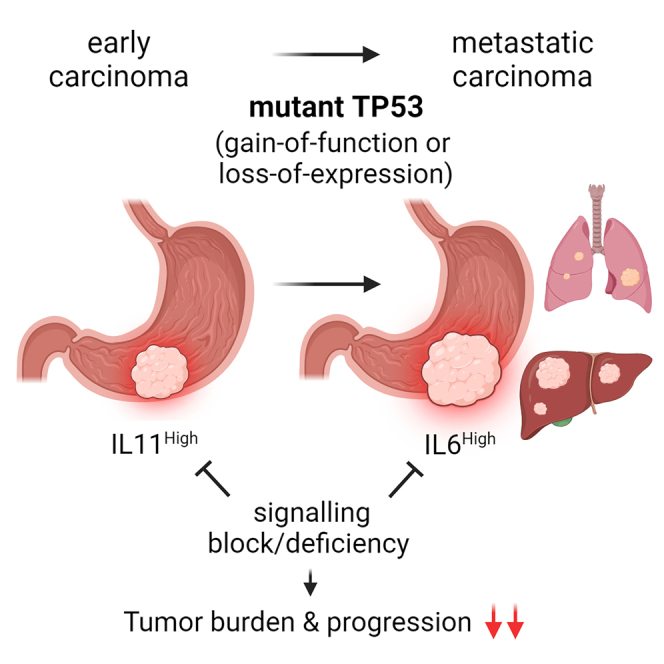

## Teaser

Huber et al. demonstrate that gain-of-function and loss-of-expression TP53 mutations drive gastric disease progression to aggressive carcinomas with synchronous metastasis. While STAT3 signaling remains rate limiting throughout gastric cancer progression, its cytokine dependency switches from IL-11 to IL-6 in response to the TP53 mutations observed during the transition to carcinomas.

## Introduction

Gastric cancer (GC) accounts for the fifth most diagnosed and third most common cause of cancer-related death worldwide.^1^ Because a majority of GC is first diagnosed when patients present with distal metastasis, the overall 1-year survival for patients with GC remains below 25%.^2^ With potentially curative surgery often not possible for patients with metastatic GC, current therapies have only limited life-prolonging effects. In part, this is due to the heterogeneity of GC at the adenocarcinoma stage, where many somatic mutations drive mutagenesis and disease progression.

Large whole-genome sequencing studies suggested specific gene mutation frequencies and allowed for the definition of molecular-based GC subtypes,^3^^,^^4^^,^^5^ consistent with the histological Lauren classification system categorizing GC in diffuse, intestinal, and mixed subtypes. While alterations in the gene encoding tumor suppressor protein (*TP53*) account for the most common mutations across all molecular subtypes, the chromosomally unstable (CIN) subtype contributes half of all GC. In addition to *TP53* mutations, the CIN subtype is often associated with the amplification of receptor tyrosine kinases (RTKs) and associated Ras signaling and, to a lesser extent, with PIK3CA pathway alterations. Unlike the most common forms of colon cancer, where the availability of early-stage, non-invasive lesions enabled the establishment of the sequence of genetic events underpinning disease, the sequence of mutations resulting in invasive GC is less clear. In particular, the requirement for activating mutations in the canonical WNT signaling pathway, including loss-of-function mutations in its primary negative regulator, the APC tumor suppressor gene, during the ontogeny of GC, remains controversial. While *APC* mutation associated with excessive activation of canonical WNT signaling accounts for the initiating event in a large majority of the most common sporadic forms of colon cancer, they occur in less than 10% of GCs. However, gene signatures associated with excessive activation of the WNT/β-catenin signaling pathway are associated with 80% of GC.^6^ On the other hand, mutations in TP53 account for relatively late events in most epithelial malignancies and are often placed at the stage when adenocarcinomas acquire aggressive metastatic characteristics.

Mutations in the *TP53* protein have been proposed to promote tumor progression as a consequence of three potentially overlapping outcomes. Besides the complete loss of function, expression of mutant proteins occurs. They predominantly arise from missense mutations in hotspots located in the DNA-binding domain of the protein, thereby impacting the formation of the transcriptionally active TP53 tetramer. Accordingly, many mutant forms of TP53 are likely to exert a dominant-negative function on wild-type (WT) TP53, while others, including the most prevalent *R175H* missense mutation in human TP53 (equivalent to *Trp53*^*R172H*^ in mice^7^^,^^8^), may result in gain-of-function consequences, as they exacerbate invasion and metastasis.^9^ However, with either type of mutation occurring in one allele, the WT allele is frequently lost through the deletion of large chromosomal fragments, resulting in loss of heterozygosity (LOH) and associated functional balancing of the remaining WT TP53 protein.^10^

Due to its role as the guardian of the genome, it has been suggested that TP53 mutations may result in vulnerabilities of cancer cells that can be exploited therapeutically, including the appearance of tumor neo-antigens.^11^^,^^12^^,^^13^ On the other hand, TP53-mutation-dependent transcriptional changes within tumor cells may also lead to “addictions” to non-mutated signaling pathways. Notably, WT TP53 is a transcriptional suppressor of interleukin (IL)-6,^14^^,^^15^ while the presence of either gain- or loss-of-function TP53 mutations increased IL-6 expression and activation of the associated signaling pathway, comprising the shared GP130 receptor subunit and STAT3 as the transcriptional signaling node.^16^^,^^17^^,^^18^ However, early adenoma stages are already fueled by aberrant STAT3 activity as a result of an oversupply of inflammatory cytokines, which is often observed even in the absence of overt gastritis.^19^ Indeed, the GP130 family cytokine IL-11, rather than IL-6, becomes rate limiting for the growth of intestinal-type GC, at least during adenomatous stages in autochthonous mouse models.^20^^,^^21^^,^^22^^,^^23^ On the other hand, elevated STAT3 in the stromal cells of the host confers an immune-suppressed tumor microenvironment, with specific roles identified for IL-6 and IL-11. While the former cytokine helps with setting up a premetastatic niches,^24^ signaling from the latter suppresses the activity of CD4 cells and antagonizes the host’s anti-tumor immune response.^25^ Meanwhile, high STAT3 activity in human patients correlates with GC progression, metastasis, and poor patient survival.^26^

Here, we provide an allelic series of autochthonous models for metastasizing intestinal-type GC that occurs in the absence of activating mutations in the WNT pathway. We identify a critical role for TP53 mutations, irrespective of their functional consequences, in the transition between non-invasive adenomas to metastasizing carcinomas. This functionally correlates with a switch from IL-11 to IL-6 dependency. Surprisingly, the requirement for IL-6 remains intrinsic to cancer cells and transplantable via the corresponding tumor organoids, thereby highlighting opportunities to discover therapeutic vulnerabilities over and above the addiction to IL-6 signaling identified here.

## Results

### *Kras*, *Pik3ca*, and *Trp53* mutations drive invasive and metastatic STAD independent of aberrant canonical WNT signaling

Because mutations in multiple common oncogenes and tumor suppressors underpin aberrant activity of signaling pathways that contribute to GC progression,^3^^,^^4^^,^^5^^,^^27^ we re-analyzed TCGA stomach adenocarcinoma (STAD) dataset for the 10 most frequently involved pathways^27^ (Figure S1A). We identified the cell cycle as being the most frequent subject of mutations, followed by alterations to the RTK/RAS, TP53, and PI3K/PTEN pathways, where simultaneous mutations within the latter three proteins occurs in 15.1% of patients with STAD (Figure S1B). Since aberrant pathway activity can occur independently of mutations in the corresponding genes, we confirmed that 48.3% of patients with STAD display simultaneous elevated transcriptional activation in the RTK/KRAS and PI3K pathways and 23% in the RTK/KRAS, PI3K, and TP53 pathways (Figures S1C and S1D). We also noted that 37.3% of all patients with STAD showed mutations in the canonical WNT signaling pathway (Figure S1A).

**Figure 1:**
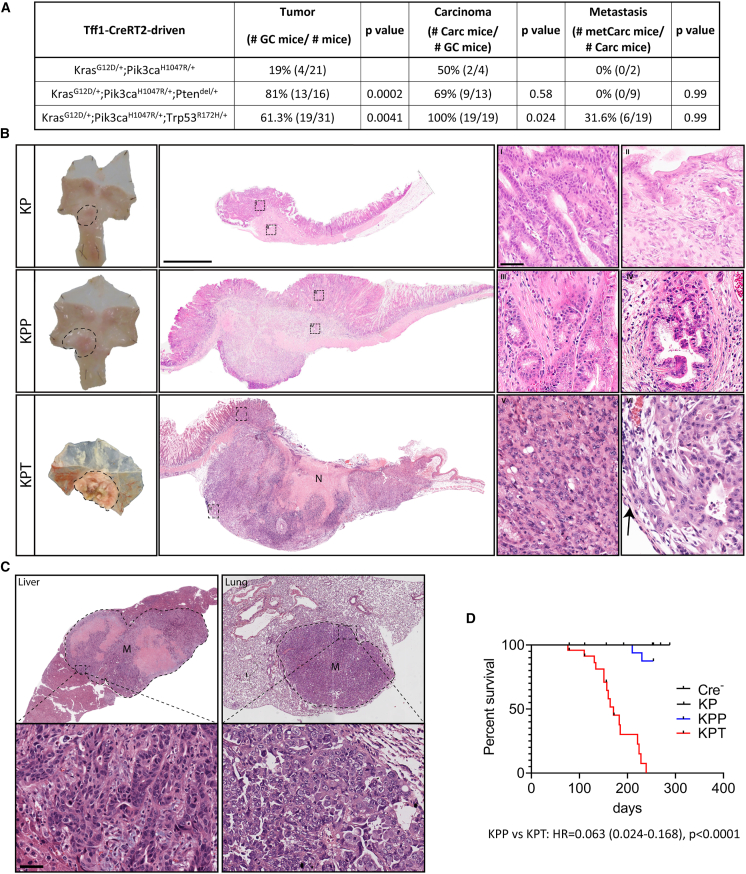
Mutant Kras, Pik3ca, and Trp53 drive gastric invasive Carc formation (A) Table shows the incidence rate in percentage and the number of mice (in brackets) with gastric tumors (GC), carcinomas (Carc), and metastatic Carc (metCarc) after tamoxifen administration to *Tff1*^*CreERT2*^-positive mice harboring the lox-STOP-lox (LSL)-flanked exons *Kras*^*LSL-G12D/+*^;*Pik3ca*^*LSL-H1047R/+*^ (*KP*), *Kras*^*LSL-G12D/+*^;*Pik3ca*^*LSL-H1047R/+*^;*Pten*^*flox/+*^ (*KPP*), or *Kras*^*LSL-G12D/+*^;*Pik3ca*^*LSL-H1047R/+*^;*Trp53*^*LSL-R172H/+*^ (*KPT*). *p* values of Fisher’s exact test are shown. (B) Representative images of whole-mount stomachs (left) and microscopic images of H&E-stained stomach cross-sections (scale bar: 2 mm) containing lesion from mice with genotypes as in (A). (I)–(VI) show higher-power images of mucosal and submucosal (=invasive) parts of the tumors (scale bar: 50 μm). N depicts necrotic tumor tissue, and the arrow (in VI) points at tumors cells invading stomach serosa. *KP* and *KPP* mice were euthanized and analyzed at 260 days post-tamoxifen administration (experimental endpoint without sickness). The *KPT* mouse was euthanized at 134 days when it reached ethical endpoints. (C) Representative H&E-stained liver (left) and lung (right) sections containing metastasis in *KPT* mice (scale bar: 50 μm). Images shown are from two mice. (D) Kaplan-Meier survival analysis of mice with genotypes: *Tff1*^*CreERT2*^-negative (*Cre*^*−*^) and *Tff1*^*CreERT2*^-positive mice harboring the mutant allele combinations: *Kras*^*LSL-G12D/+*^;*Pik3ca*^*LSL-H1047R/+*^ (*KP*), *Kras*^*LSL-G12D/+*^;*Pik3ca*^*LSL-H1047R/+*^;*Pten*^*flox/+*^ (*KPP*), or *Kras*^*LSL-G12D/+*^;*Pik3ca*^*LSL-H1047R/+*^;*Trp53*^*LSL-R172H/+*^ (*KPT*). For the *KPP* versus *KPT* comparison, the hazard ratios (HRs) and *p* values were calculated with log-rank (Mantel-Cox) test are displayed. *n* = 16, 16, 16, and 24, respectively. See also Figure S1 and Table S1.

To establish corresponding mouse models, we exploited our bacterial articial chromosome (BAC)-transgenic and tamoxifen-inducible *Tff1:CreERT2* driver strain^28^ to conditionally induce various combinations of latent activatable alleles to encode KRAS^G12D^, PIK3ca^H1047R^, or TP53^R172H^ mutant proteins, alongside the deletion of PTEN following induction of the *Pten*^flox^ allele. While we previously described that gastric-epithelial-specific expression of *Kras*^*G12D*^ is sufficient to trigger gastric adenoma formation,^28^ we only detected adenomas when concurrently mutating *Pi3kca* and *Pten* with the *Tff1*^*CreERT2*^ driver stain, not when either gene was mutated individually (Figures S1E–S1H). Meanwhile, 19% of tamoxifen-induced compound mutant *Tff1*^*CreERT2*^;*Kras^LSL-^*^*G12D*^^*/+*^;*Pik3ca^LSL-^*^*H1047R*^^*/+*^ (referred to as *Tff1*^*CreERT2*^;*Kras*^*G12D/+*^;*Pik3ca*^*H1047R/+*^ or *KP*) mice developed gastric tumors, of which 50% presented as adenocarcinomas (Figure 1A). However, further augmenting PI3K pathway activation through heterozygous ablation of *Pten* in triple-mutant *Tff1*^*CreERT2*^;*Kras^LSL-^*^*G12D*^^*/+*^;*Pik3ca^LSL-^*^*H1047R*^^*/+*^;*Pten*^flox/+^ (referred to as *Tff1*^*CreERT2*^;*Kras*^*G12D/+*^;*Pik3ca*^*H1047R/+*^;*Pten^del/+^* or *KPP*) mice increased the overall frequency of gastric tumors to 81%, and over two-thirds of the tumors in *KPP* mice had progressed to carcinomas. Interestingly, the lesions in neither *KP* nor *KPP* mice progressed to metastatic stages (Figures 1A and 1B). Indeed, despite the larger size of *KPP* tumors when compared to their *KP* counterparts, both types of tumors are located in the antrum of the stomach. Either type of tumor retains the intestinal subtype appearance and remains characterized by elongated pits alongside enlarged glandular structures associated with an accumulation of intraepithelial lymphocytes, while submucosal invasion was more evident in *KPP* tumors (Figure 1B).

Next, we challenged *KP* mice with additional TP53 mutations to replicate a common event associated with tumor progression in patients with STAD. Indeed, simultaneous gene (in)activation in the resulting *Tff1*^*CreERT2*^;*Kras^LSL-^*^*G12D*^^*/+*^;*Pik3ca^LSL-^*^*H1047R*^^*/+*^;*Trp53^LSL-^*^*R172H*^^*/+*^ (referred to as *Tff1*^*CreERT2*^;*Kras*^*G12D/+*^;*Pik3ca*^*H1047R/+*^;*Trp53*^*R172H/+*^ or *KPT*) mice transformed the lesions observed in *KPP* mice to adenocarcinomas of the intestinal type. *KPT* tumors were classified as poorly differentiated tubular-type adenocarcinomas and showed extensive lymphatic and submucosal invasion, which was frequently associated with tissue necrosis (Figures 1A, 1B, and S1I; Table S1). *KPT* carcinomas are predominantly located in the antrum of the stomach but can also extend across the entire glandular stomach leading into the gastroesophageal junction (Figure 1B). One-third of tumor-bearing, moribund *KPT* mice presented with liver and lung metastases (Figures 1A–1C and S1J), which correlated with reduced overall survival when compared to mice from the *KPP* and *KP* cohorts (Figure 1D).

Given the high prevalence of mutations in the canonical WNT signaling pathway in human GC, we excluded contributions by secondary serendipitous mutations in this pathway to tumor formation in *KPP* and *KPT* mice using the absence of nuclear β-catenin as a surrogate marker for activation of the canonical WNT pathway (Figure S2A). Likewise, we could not detect activation of the canonical WNT target and stem cell genes *Lgr5* and *Sox9* but noted increased expression of the more promiscuous genes *CD44*, *Ccnd1*, and *Myc* in *KPT* tumors (Figure S2B), which have also been identified as targets for STAT3.

### Loss-of-function and gain-of-function *Trp53* mutations drive aggressive metastatic disease

**Figure 2:**
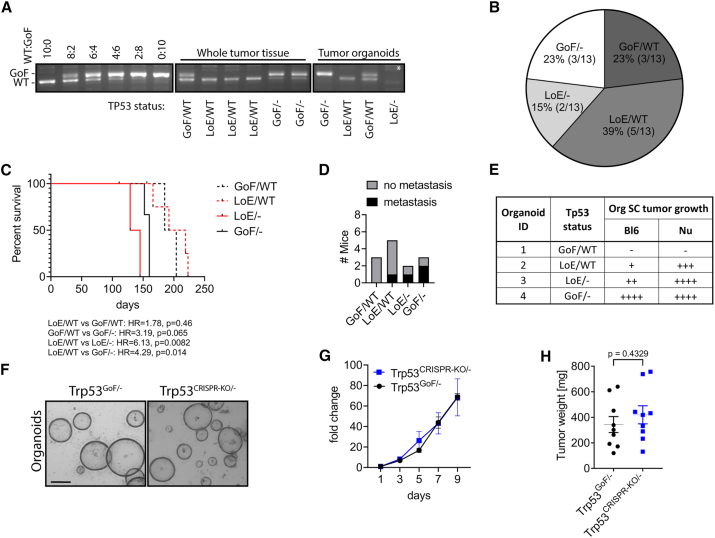
Tp53 mutation status in KPT tumors (A) Image showing the *Trp53* status PCR assay performed on genomic DNA of whole-tumor or organoid (Org) lysates from *Tff1*^*CreERT2/+*^;*Kras*^*LSL-G12D/+*^;*Pik3ca*^*LSL-H1047R/+*^;*Trp53*^*LSL-R172H/+*^ (*KPT*) mice. The upper band indicates the presence of the GoF allele and the lower band the WT allele. GoF, gain of function = recombined *Trp53*^*LSL-R172H*^ allele; LoE, loss of expression = non-recombined *Trp53*^*LSL-R172H*^ allele leads to no *Trp53* being expressed; WT, wild-type *Trp53*, − = the wild-type *Trp53* allele being genetically lost. Asterisk (^∗^) indicates that secondary genomic DNA PCR was used to confirm the presence of the non-recombined *Trp53*^*LSL-R172H*^ allele. (B) Frequency of *Trp53* status assessed by PCR assay (shown in A) in tumors from *KPT* mice (*n* = 13). (C) Kaplan-Meier survival analysis of *KPT* mice based on tumor *Trp53* status. The HRs and *p* values of log-rank (Mantel-Cox) test are shown. *n* = 3, 5, 2, and 3, respectively. (D) Number of *KPT* mice presenting with or without metastasis based on primary tumor *Trp53* status. (E) Table depicting the tumor allograft growth potential of GC Orgs derived from *KPT* mice with indicated *Trp53* status in C57BL/6 WT and BALB/c Nu/Nu host mice (*n* = 4 host mice per Orgs per background). −, no tumor growth within 80 days; +, initial tumors ≤50 mm^3^ form but do not progress within 80 days; ++, tumors grow ≥1,000 mm3 within 80 days; +++, tumors grow ≥1,000 mm3 within 55 days; ++++, tumors grow ≥1,000 mm3 within 30 days. (F) Representative photomicrograph of parental (P) *Trp53*^*GoF/−*^ Orgs and one of the co-isogenic CRISPR-Cas9-mediated *Trp53*^*CRISPR-KO/−*^ daughter Org clone (scale bar: 200 μm). (G) *In vitro* Org growth assessment of *Trp53*^*GoF/−*^ P and *Trp53*^*CRISPR-KO/−*^ daughter Orgs. Average fold changes (relative to day 1) of luminescence of *n* = 3 independent experiments are shown; each experiment was conducted with four technical replicates. Data represent mean ± SEM. (H) Tumor mass at endpoint of *Trp53*^*GoF/−*^ Orgs and *Trp53*^*CRISPR-KO/−*^ daughter Orgs subcutaneously (s.c.) injected into C57BL/6 host mice (*n* = 9 and 9). Two-sided, unpaired t test *p* values and mean ± SEM are shown. See also Figure S3.

TP53 mutations can be classified to confer either loss-of-function or possible gain-of-function consequences.^9^ Although the direct relationship between specific amino acid substitutions in *Tpr53* and functional outcome remains controversial, LOH of the remaining WT allele is a frequent consequence. Prior to Cre-mediated recombination, the lox-STOP-lox cassette within intron 1 of the targeted *Trp53*^*R172H*^ locus blocks its expression, thereby resulting in a “loss-of-expression” allele (subsequently referred to as *Trp53*^*LoE*^).^8^ However, upon Cre-mediated recombination, this allele is reconstituted to contain the R172H substitution, which corresponds to the R175H hotspot gain-of-function mutations found in human patients with cancer (subsequently referred to as *Trp53*^*GoF*^) (Figure S3A). Due to the incomplete activity of Cre recombinase and the aforementioned LOH observations, we clarified the *Trp53* status in gastric carcinomas and tumor organoids established from *KPT* mice (Figures 2A and S3B). We detected all four possible variants of *Trp53* allele combinations, without any apparent preference for one status over another (Figure 2B). Strikingly, *Trp53*^*LoE/−*^ or *Trp53*^*GoF/−*^ tumor-bearing mice showed reduced survival when compared to littermates with *Trp53*^*LoE/WT*^ or *Trp53*^*GoF/WT*^ tumors that still harbored a WT allele (Figure 2C). Meanwhile, primary tumors of mice with synchronous metastases returned all but the *Trp53*^*GoF/WT*^ allele combination in their primary lesions (Figure 2D).

In order to address whether these allele combinations functionally contributed to the growth characteristics of primary *KPT* tumors, we established organoid cultures from the corresponding primary tumors to cover all four allele combinations. Upon subcutaneous transplantation these organoids form tumor allografts, which histologically resemble the stomach carcinomas they have been established from (Figure S3C). We investigated the organoids’ growth characteristics when grown as allograft tumors in either immune-competent C57BL/6J WT mice or immune-deficient BALB/c nude mice (Figure 2E). Strikingly, allografts consistently grew the quickest when established from tumor organoids that lacked a *Trp53*^*WT*^ allele and the slowest when the altered allele was still balanced with its WT counterpart, irrespective of the immune status of the host. To further investigate whether the putative gain-of-function TP53 mutations may affect tumor growth differently compared to loss-of-expression TP53 mutations, we used CRISPR-Cas9 gene editing to functionally inactivate the *Trp53*^*Go*^^*F*^ allele in *Trp53*^*GoF/−*^ organoids to yield a co-isogenic *Trp53*^*CRISPR-KO/−*^ (KO, knockout) organoid clone (Figure S3D). However, neither organoid growth *in vitro* (Figures 2F and 2G) nor allograft tumor growth in immune-competent host mice was different between the clonal *Trp53*^*GoF/−*^ parental and *Trp53*^*CRISPR-KO/−*^ daughter organoids (Figures 2H and S3E). We surmise from these observations that both loss-of-expression and *Trp53*^*R172H*^-encoded putative gain-of-function mutations promote tumor growth and progression and that this is further exaggerated through the loss of the remaining *Trp53*^*WT*^ allele.

### Mandatory STAT3 activity in tumor cells undergoes a TP53-dependent switch from IL-11 to IL-6

**Figure 3:**
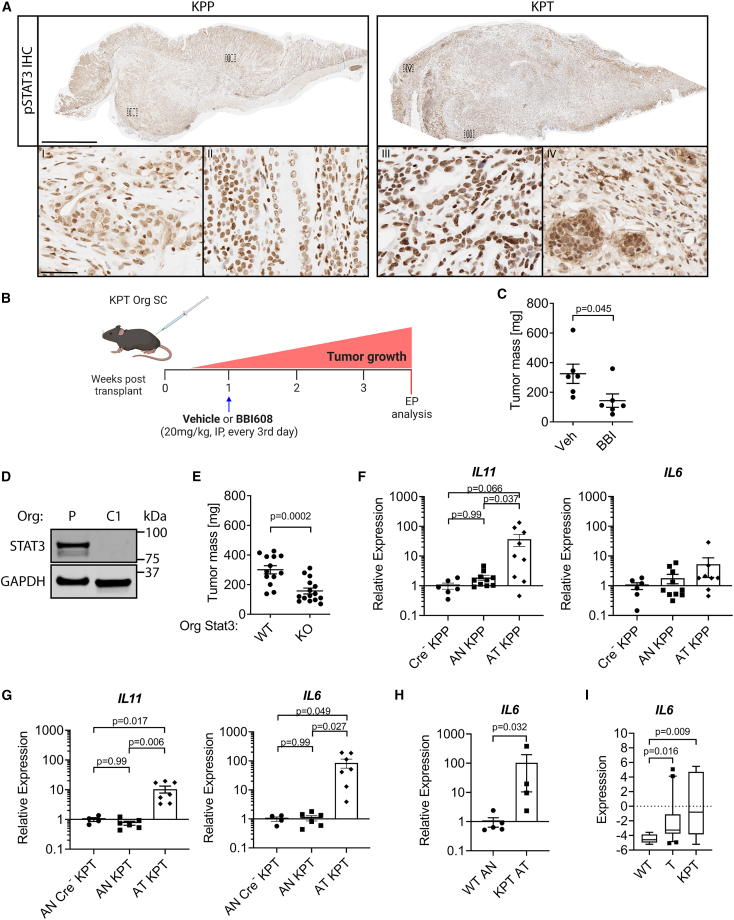
Stage-specific ligand switch for mandatory Stat3 activity in tumor cells (A) Representative photograph of pSTAT3 immunohistochemistry staining of *Tff1*^*CreRT2*^;*Kras*^*LSL-G12D/+*^;*Pik3ca*^*LSL-H1047R/+*^;*Pten*^*flox/+*^ (*KPP*; left) and *Tff1*^*CreRT2*^;*Kras*^*LSL-G12D/+*^;*Pik3ca*^*LSL-H1047R/+*^;*Trp53*^*LSL-R172H/+*^ (*KPT*; right) mouse stomachs (scale bar: 2 mm) with magnification of the invasive tumor front (I and III) and the mucosal tumor core (II and IV) (scale bar: 50 μm). (B) Schematic of *KPT* mutant GC Org s.c. transplantation into C57BL/6 WT mice with indicated treatment cohorts. EP, endpoint. Created with BioRender.com. (C) Tumor mass at endpoint of *KPT* Org SC allograft experiment as outlined in (B) for vehicle (Veh)- and BBI608 (BBI)-treated animals. *n* = 6 and 6 (experiment was performed once). (D) Immunoblotting for STAT3 and GAPDH protein on Org lysates from P and CRISPR-Cas9 *Stat3*^*KO*^ clone 1 (C1). (E) Tumor mass at endpoint of *Stat3*^*WT*^ (WT) or *Stat3*^*KO*^ (KO) *KPT* GC Orgs following s.c. implantation into C57BL/6 mice. *n* = 13 and 15 (pooled data from two independent experiments). (F and G) qPCR gene expression analysis for *Il11* and *Il6* in whole-tissue lysates of antrum normal (AN) tissue and antrum tumor (AT) of indicated genotypes. Expression data are presented relative to the mean of *Cre*^*−*^*KPP* (F) or AN *Cre*^*−*^*KPT* (G) data points. *p* values shown are from one-way ANOVA + Tukey’s multiple comparison testing; for the *IL6* graph in (F), all *p* > 0.3. *n* = 6, 10, and 9 (F, left) and *n* = 6, 10, and 8 (F, right). *n* = 4, 6, and 7 (G, both graphs). (H) qPCR-determined expression levels of *IL6* in Orgs derived from WT antrum stomach and stomach tumors of *KPT* mutant mice. Expression data are presented relative to WT AN values. *p* values of Mann-Whitney test are shown. *n* = 5 and 4. (I) Expression levels of *IL6* in human gastric cancer cell lines grouped into *TP53*^*WT*^ (WT), *TP53* (T) mutant, or *KRAS*;*PI3K*;*TP53* (*KPT*) mutant activation signature positive. RNA sequencing data were downloaded from the Broad Institutes Cancer Cell Line Encyclopedia. Data are shown as box and whiskers plots (10th–90th percentile). *n* = 8, 21, and 7, respectively. The y axis depicts log2 counts per million values. Kruskal-Wallis and Dunn’s multiple comparison’s test were performed. Data represent mean ± SEM (C and E–I). Each symbol represents a biological replicate, specifically one mouse (C and E–G), independent Org culture (H), or human GC cell line (I). Two-sided Student’s t test *p* values are shown (C and E). See also Figure S4.

We and others have previously demonstrated that STAT3 signaling in response to IL-6 family cytokines provides a rate-limiting signal for gastrointestinal tumors that arise from *bona fide* oncogenic driver mutations, including in *APC*, *KRAS*, or other genes.^20^^,^^21^^,^^22^^,^^23^^,^^29^ Indeed, using the nuclear presence of the phosphorylated STAT3 (pSTAT3) isoform as a surrogate marker, we identified active STAT3 signaling in epithelial cells localized in the mucosa as well as the submucosal invasive fronts across gastric tumors of *KPP* and *KPT* mice (Figure 3A), while in unaffected normal antrum, only a proportion of the epithelial cells stained positive for pSTAT3 (Figure S4A). To functionally evaluate this observation, we performed subcutaneous allografts with organoids derived from the invasive fronts of adenocarcinomas from *KPT* mice (Figure 3B). Treatment with the small-molecule STAT3 inhibitor BBI608^30^ significantly reduced tumor size, suggesting that the growth of *KPT* mutant tumors is fueled by STAT3 (Figures 3C and S4B). We genetically confirmed that the effect of systemic BBI608 administration could, at least in part, be due to tumor-cell-intrinsic STAT3 signaling because CRISPR-Cas9-mediated STAT3 deletion in *KPT* organoids (Figure 3D) reduced their growth when established as allografts in *Stat3*^*WT*^ hosts (Figure 3E). Because the *in vitro* growth characteristics between STAT3-proficient and STAT3-deficient *KPT* organoids remained indistinguishable in the absence of exogenously added gp130 family cytokines (Figures S4C and S4D), we surmise that Stat3 deletion reduces the *in vivo* tumorigenicity rather than merely reducing tumor cell proliferation.

We next aimed to identify the contribution of individual GP130 cytokines to the growth of *KPP* and *KPT* allograft tumors *in vivo*. *IL11* expression was increased 40-fold in *KPP* tumors when compared to adjacent non-tumor epithelium, while *IL6* expression remained comparable between these two compartments (Figure 3F). By contrast, *IL6* expression was elevated 85-fold in *KPT* tumors, while the IL-11 expression increase was limited to 10-fold (Figure 3G). Importantly, *IL6* expression was elevated in *KPT* organoids (Figure 3H), reminiscent of the increased IL-6 expression in patient-derived human TP53-mutant and *KPT* GC cell lines (Figure 3I). Indeed, co-culture of bone marrow-derived macrophages (BMDMs) with *KPT* organoids increased *IL6* expression as long as the *KPT* organoids harbored a mutated *Trp53* allele (Figure S4E). The presence of the *Trp53*-mutant alleles was consistently associated with elevated *IL6*, but not *IL11*, expression in *KPT* organoids, human GC cell lines, and BMDM-organoid co-cultures (Figures S4F–S4H). In addition, *KPT* tumor organoids showed increased IL-6 receptor (*IL6r*) expression, while expression of their IL-11 receptor (*IL11r*) remained unaltered (Figure S4I). These results suggest that the elevated IL-6 associated with TP53-mutant tumors may be derived from the tumor cells as well as from macrophages and other cells of the microenvironment and that TP53-mutant GC tumor cells develop an IL-6 dependency to serve their requirement for continuous STAT3 signaling.

**Figure 4:**
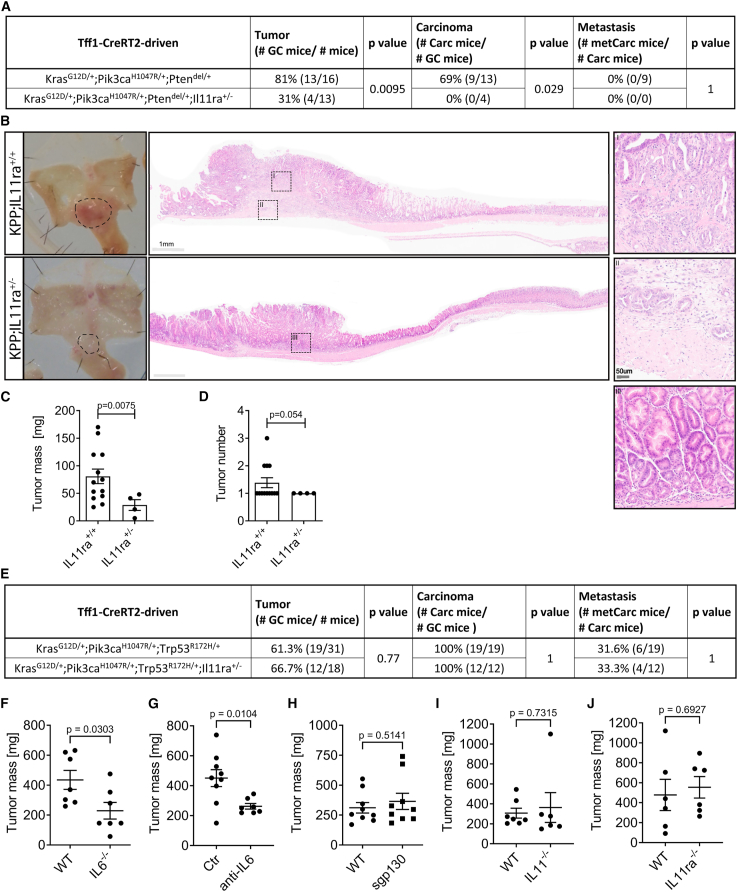
Functional stage-specific reliance on IL-6 family cytokines (A) Table summarizing gastric tumor (GC), Carc, and metCarc incidences after tamoxifen administration to *Tff1*^*CreRT2*^-positive mice either harboring *Kras*^*LSL-G12D/+*^;*Pik3ca*^*LSL-H1047R/+*^;*Pten*^*flox/+*^ (*KPP*) or *Kras*^*LSL-G12D/+*^;*Pik3ca*^*LSL-H1047R/+*^;*Pten*^*flox/+*^;*Il11ra*^+/−^ (*KPP*;*IL11ra*^+/−^). *KPP* data are also shown in Figure 1A. (B) Representative H&E staining of whole-mount stomachs and microscopic images of stomach lesions and mice with genotypes as in (A). Scale bars are as indicated. (C and D) Gastric tumor burden (C) and tumor number (D) analysis is shown from tumor-bearing mice (*Tff1*^*CreERT2*^;*Kras*^*LSL-G12D/+*^;*Pik3ca*^*LSL-H1047R/+*^;*Pten*^*flox/+*^) harboring either *IL11ra*^+/+^ or *IL11ra*^+/−^ at 250 days post-mutant allele induction. *n* = 13 and 4 (C and D). (E) Table summarizing gastric tumor (GC), Carc, and metCarc incidences after tamoxifen administration to *Tff1*^*CreRT2*^-positive mice harboring either *Kras*^*LSL-G12D/+*^;*Pik3ca*^*LSL-H1047R/+*^;*Trp53*^*LSL-R172H/+*^ (*KPT*) or *Kras*^*LSL-G12D/+*^;*Pik3ca*^*LSL-H1047R/+*^;*Trp53*^*R172H/+*^;*Il11ra*^+/−^ (*KPT*;*IL11ra*^+/−^). *KPT* data are also shown in Figure 1A. (F–J) Tumor mass at endpoints of s.c. Org allograft experiment where *KPT* GC Orgs were transplanted into either WT or *IL-6*^−/−^ hosts (F). WT hosts were treated when tumors reached 100 mm^3^ volume with isotype control antibody (Ctr) or with IL-6 neutralizing antibody (anti-IL-6) (G). Orgs were implanted into WT or *sgp130* hosts (H), WT or *IL11*^−/−^ hosts (I), and WT or *IL11ra*^−/−^ hosts (J). Experiments were performed once. *n* = 7 and 7 (F), 9 and 7 (G), 9 and 9 (H), 7 and 6 (I), and 6 and 6 (J). Data represent mean ± SEM (C, D, and F–I). Each symbol represents a biological replicate, specifically one mouse (C, D, and F–J). *p* values of Fisher’s exact test (A and E) and two-sided Student’s t test (C, D, and F–J) are shown. See also Figure S5.

To functionally clarify whether IL-11-mediated Stat3 signaling contributes to the formation and progression of gastric tumors, we generated *KPP*;*IL11ra*^+/−^ mice because we had previously shown that monoallelic ablation of the IL-11Ra receptor subunit impaired the formation of signaling-competent IL-11:IL-11Ra:gp130 receptor complexes.^21^ Indeed, the size and overall tumor frequency in *KPP*;*IL11ra*^+/−^ mice was reduced when compared to *KPP*;*IL11ra*^+/+^ littermates, and tumors in *KPP*;*IL11ra*^+/−^ mice failed to progress to carcinoma stages (Figures 4A–4D). In stark contrast, the incidence of carcinoma, their depth of invasion, and their metastatic capacity remained comparable between *Trp53*-mutant *KPT*;*IL11ra*^+/−^ and *KPT*;*IL11ra*^+/+^ littermates (Figures 4E and S5A). Consistent with this observation, pSTAT3 protein levels and nuclear staining were decreased in tumors from *KPP*;*IL11ra*^+/−^ compared to those from *KPP*;*IL11ra*^+/+^ mice but remained comparable between tumors of *KPT*;*IL11ra*^+/−^ and *KPT*;*IL11ra*^+/+^ mice (Figures S5B–S5E). Since we detected increased *IL6* expression in the tumors of *KPT* mice, we also assessed the causal consequences of this correlation and found that *KPT* organoid tumor allografts grew slower in IL-6-deficient hosts (Figure 4F). Likewise, therapeutic administration of neutralizing IL-6 antibodies to WT hosts with established *KPT* tumor allografts reduced their growth (Figure 4G). Neither therapeutic IL-6 inhibition nor IL-6 cytokine deficiency in the host environment led to a significant increase in CD8 T cell tumor infiltration (Figures S5F–S5H). We confirmed that the IL-6 signaling dependency did not rely on IL-6 *trans*-signaling, as blocking IL-6 *trans*-signaling in *sgp130* hosts did not alter tumor growth compared to IL-6 *trans*-signaling-proficient WT hosts (Figure 4H). Likewise, tumor growth was also not suppressed when *KPT* organoids were implanted in either IL-11 ligand- or IL-11Ra receptor-deficient hosts (Figures 4I and 4J). Collectively, our data established tumor-intrinsic STAT3 signaling as a rate-limiting gatekeeper function for *KPT* tumors and suggest that the acquisition of Trp53 mutations induces a switch from IL-11 dependency to IL-6 dependency.

### Elevated IL-6 and STAT3 signaling predicts poor survival in patients with gastric adenocarcinoma

**Figure 5:**
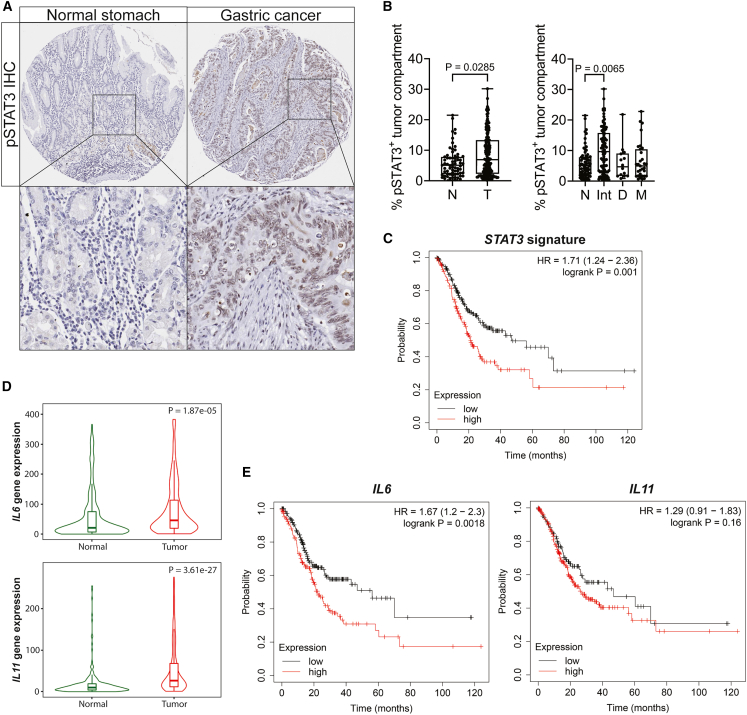
IL-6-IL-11-Stat3 signaling in patients with gastric cancer (A) Representative image of pSTAT3 immunohistochemical staining on normal stomach and gastric cancer cores of tumor tissue microarray. (B) HALO quantification of pSTAT3 positivity specifically in the tumor compartment comparing normal (N) versus gastric tumor (T) and normal tissues versus gastric cancer Lauren subtypes (right graph; Int, intestinal; D, diffuse; M, mixed). Each symbol represents a biological replicate, specifically individual patient samples. *n* = 68 and 154 (left) and 68, 95, 14, and 31 (right). (C) Kaplan-Meier survival analysis (overall survival) of STAT3 signaling activation gene signature (*STAT3*, *SOCS3*, *OSMR*, *CLDN12*, *PIM3*) high versus low in stomach adenocarcinoma (prepared with Kaplan-Meier plot). *n* = 220 (low) and 151 (high); stratified with KMplot’s “best cutoff” algorithm; false discovery rate (FDR) = 20%. (D) *IL6* and *IL11* mRNA expression in normal stomach (N) versus gastric tumor (T) tissues. *IL6* expression is 1.86-fold and *IL11* is 3-fold increased in the stomach adenocarcinoma (median fold change). *p* values from Mann-Whitney test (prepared with TNMplot). (E) Kaplan-Meier survival analysis (overall survival) of *IL6* and *IL11* high versus low RNA expression in stomach adenocarcinomas (prepared with KMplot). *n* = 218 and 153 (left; FDR = 20%) and 132 and 239 (right; FDR = 100%); stratified with KMplot’s “best cutoff” algorithm. Data represent mean ± SEM (B) and a violin plot with inner box plot depicting 1st to 3rd quartile and median (D). *p* values of Mann-Whitney test (B, left, and D), Kruskal-Wallis + Dunnett’s multiple comparisons test (B, right), and HRs and *p* values of log-rank (Mantel-Cox) test (C and E) are shown. See also Figure S6 and Table S2.

In order to clinically translate the causal relationship between elevated STAT3 signaling activity and tumor progression in our murine *KPP* and *KPT* models, we interrogated STAD patient samples for evidence of STAT3 activity (Table S2). We used the HALO software for unbiased calls between tumor and non-neoplastic surrounding epithelial/stromal cell compartments across tissue arrays containing normal gastric and adenocarcinoma tissues (Figures S6A and 5A). We detected the strongest pSTAT3 signal in the tumor compartment of intestinal-type GC biopsies (Figure 5B). However, when quantifying whole tissues across tumor and stromal compartments, we found comparable pSTAT3 between normal and tumor core biopsies across histological subtypes of GC (Figure S6B).

To independently confirm these observations, we took advantage of our STAT3 gene signature comprising the *bona fide* STAT3-target genes *STAT3*, *SOCS3*, *CLDN12*, *OSMR*, and *PIM3*, which we developed from our previous STAT3 chromatin immunoprecipitation sequencing and RNA sequencing data of tumors recovered from *gp130*^*F/F*^ mice stimulated with gp130 cytokines.^31^ When assigning TCGA-patient specimens according to their expression signature into STAT3^Low^ and STAT3^High^ cohorts, Kaplan-Meier survival probability analysis revealed a poorer outcome for the latter (Figures 5C and S6C). In turn, specimens of the STAT3^High^ patient cohort also revealed higher expression of IL-6 or IL-11 when compared to the STAT3^Low^ cohort (Figure S6D). Although GC specimen displayed elevated expression of both *IL6* and *IL11*, when compared to normal stomach tissue (Figure 5D), we found that, akin to our observations in *KPP* and *KPT* tumors in mice, *IL6*, rather than *IL11*, expression correlated with poor patient survival (Figures 5E and S6E). Thus, the gatekeeper role for the IL-6-dependent STAT3 signaling cascade for advanced GC is conserved between preclinical mouse models and human GC, thereby elevating ligand-specific activation of the GP130/STAT3 signaling cascade as a potential, stage-specific therapeutic target (Figure S7).

## Discussion

Here, we create an allelic series of compound mutant mice to enable the inducible formation of autochthonous metastatic tumors in the glandular epithelium of the mouse stomach to replicate the accumulation of some of the most frequently observed mutations in patients with intestinal-type GC. We demonstrate that co-activation of the KRAS and PIK3CA/PTEN signaling pathways is sufficient for the development of early-stage gastric adenocarcinomas. The additional mutation in *Trp53* further drives disease progression, with all corresponding mice harboring highly invasive adenocarcinomas and frequent distal metastasis, predominantly to the liver. The involvement of these mutations is reminiscent of the progression of the human disease, where TP53 mutations are found more often in advanced GC involving liver metastasis than in advanced GC without distal metastasis^32^^,^^33^ and where TP53 mutations are associated with poor prognosis in the microsatellite-stable subtype.^4^

The reproducible formation of tumors in *KPP* and *KPT* mice helps to clarify whether there is an absolute requirement for aberrant canonical WNT signaling, which has previously been used to build a complementing series of GC mouse models based on loss-of-function mutations in the *Apc* tumor suppressor gene.^6^ Interestingly, in the absence of predetermined mutations in the canonical WNT signaling pathway in our *KPP* and *KPT* models, we did not detect nuclear localization of β-catenin, the surrogate marker for activation of canonical WNT signaling. However, in these tumors, we find elevated expression of the promiscuous WNT target genes *CD44*, *Ccnd1*, and *Myc*, consistent with their transcriptional control through converging RAS/PI3K and STAT3 signaling resulting from the introduced mutations and IL-11/IL-6 cytokine production, respectively. Indeed, this is reminiscent of the conversion of the canonical WNT and STAT3 signaling pathways in the context of mutant APC-driven tumor formation in the colonic mucosa.^29^

The various impacts of genomic alterations in TP53 result from specific mutations causing a broad range of outcomes, including the complete loss of protein function and the generation of proteins with dominant-negative functions and proteins with gain-of-function effects.^9^ Although putative gain-of function alterations, including R175H and R248Q, are found in over 20% of the most common cancers,^34^ the gain-of-function effect of these missense mutations is likely to only occur in specific cellular contexts.^35^ This may explain why recording only the overall TP53 mutation status has proven to be of little prognostic value for patients with GC.^36^^,^^37^^,^^38^^,^^39^^,^^40^ However, predictive information can be gained when stratifying patients with GC according to their molecular subtypes and the nature of their TP53 mutation,^4^^,^^33^^,^^41^^,^^42^^,^^43^ in particular when also including BCL2-associated X, neurexin 1, Yes-associated transcriptional regulator 1, and other oncogenic mutations.^38^^,^^39^^,^^40^

Owing to the design of the *Trp53*^*R172H*^ allele and our breeding strategy of *KPT* mice, functional conclusions can be drawn with respect to the presence of the latent “loss-of-expression” or recombined “gain-of-function” allele, as well as the “balancing” contribution of a *Trp53*^*WT*^ allele. Notwithstanding our inability to determine whether loss of the *Trp53*^*WT*^ allele occurs by allelic duplication of the mutant allele or other mechanisms, we find that the “loss-of-expression” and “gain-of-function” mutant alleles confer similar detrimental effects on the overall tumor burden and survival of *KPT* mice. Meanwhile, loss of a balancing *Trp53*^*WT*^ allele reduces survival, reminiscent of LOH being frequently observed at the *TP53* locus in human GC and other cancers^10^^,^^44^ and possibly owing to observations that LOH further accelerates genomic instability and cancer progression.^45^

Irrespective of the prevailing combinations of mutations in *KPP* or *KPT* mice, we discover a gatekeeper role for tumor-cell-intrinsic STAT3 signaling that provides attractive therapeutic targets, including interference with the activity of upstream cytokines. Indeed, our results expand on previous observations that excessive STAT3 activity in the gastric epithelium mediated by IL-11 triggers the formation of non-invasive adenomas in *gp130*^*F/F*^ mice.^21^ Meanwhile, IL-6, and its associated STAT3 signaling, has recently gained attention as an inflammatory cytokine facilitating the formation of a (pre)metastatic niche for solid malignancies, including breast cancer.^24^ TP53 is a transcriptional repressor of IL-6 expression,^14^^,^^15^ and loss of WT TP53 or acquisition of TP53 missense mutations increases IL-6 expression and STAT3 signaling.^16^^,^^17^^,^^18^ Insights from the switch of cytokine dependency between adenomatous *KPP* tumors expressing WT *Trp53* and metastatic *KPT* tumors harboring genomic alterations in *Trp53* suggest different cytokine requirements *in situ*, despite the quality of the intracellular signaling associated with the shared gp130 co-receptor subunit and associated STAT3 signaling remaining identical in response to IL-11 and IL-6.^21^ However, here, we observe a strong bias of *IL6* over *IL11* expression in *KPT* compared to *KPP* tumors, with a concomitant selective upregulation of IL-6Ra over IL-11Ra in response to *TP53* mutations. This suggests that the increased requirement for STAT3 signaling in tumor cells of *KPT* tumors, including the possible involvement of tumor cells that hitherto did not respond to IL-11, appears to be realized by the coordinated upregulation of IL-6 and its cognate IL-6Ra receptor subunit. While the observed switch of cytokine dependency from IL-11 to IL-6 during tumor progression is consistent with the recognized role of IL-6 as a master regulation across many cell types within the tumor microenvironment and a broadening of responsive cells through inflammation-associated IL-6 *trans*-signaling,^46^ the latter only contributes marginally to the growth of gastric organoid tumors in a subcutaneous setting.

Additional molecular mechanisms may underpin the TP53-mediated cytokine switch. For instance, RAS signaling via the AP-1 complex transcriptionally activates *IL11* gene expression in cancer cells,^47^ while KRAS-mutant cells are poised to coerce cells of the tumor microenvironment into the production of cytokines.^48^^,^^49^ Meanwhile, a super-enhancer in the *IL6* locus is susceptible to BRD4 inhibition,^50^ relevant to a recent observation that the TP53^R172H^ protein induces expression of the CSF1 cytokine via an interaction with BRD4 on a histone 3 lysine 27 acetylation-rich region.^51^ Indeed, epigenetic activation of this super-enhancer, as judged by Assay for Transposase-Accessible Chromatin (ATAC) sequencing signals, correlated with IL-6 expression in patients with esophageal and pancreatic cancers. However, TP53^R172H^ may also increase JAK2/STAT3 signaling independent of gp130 cytokines through direct interaction with the gp130-associated phosphatase SHP2,^17^ reminiscent of the direct binding of the TP53^R248Q^ protein to activated pSTAT3, thereby “short circuiting” the ligand dependency of the STAT3 signaling cascade during colon cancer progression.^18^

Although initial clinical trials with the anti-IL-6 or -IL-6Ra monoclonal antibodies siltuximab and tocilizumab, respectively, did not reveal clinical benefits in patients with cancer as a monotherapy,^52^^,^^53^ such trials have not been conducted in patients with GC, nor were patients stratified for their mutational TP53 status. Our study reveals a TP53-mutation-dependent switch from IL-11 to IL-6 to satisfy the cancer cells’ continuous need for excessive STAT3 activity. Furthermore, our data suggest that the most prominent therapeutic window for interference with IL-6 signaling may occur at the stage of initiation/early colonization of the (pre)metastatic niche while possibly providing little benefit at early-stage disease prior to somatic mutation in TP53. However, and irrespective of TP53 mutations, systemic inhibition of IL-6 signaling may also confer responsiveness to otherwise immune checkpoint blockade refractory cancers, based on the observations of Huseni et al. that high serum IL-6 correlated with dysfunctional CD8^+^ T cells in patients with renal cell carcinoma and that inhibition of IL-6 signaling alleviated the break on effector cell differentiation in tumor-bearing mice.^54^ Further mechanistic insights from the *KPT* model will help to better define recruitment criteria for future clinical trials exploring the beneficial effect of anti-IL-6 signaling therapies.

### Limitations of the study

Our conclusion of phenotypic overlap between GC burden in mice harboring a loss-of-expression (LoE) versus a potential gain-of-function (GoF) *Tpr53* allele is construed by the limited number of animals included in the study and may warrant further validation of the insights of this study.

The identified mutant TP53-associated switch from IL-11 to IL-6 dependency in GC growth should be reconciled with additional independent cellular models. While outside the scope of this study, a comprehensive investigation into the role of tumor immune cells in the cytokine dependency switch and phenotypes driven by gain/loss-of-function TP53 mutations may yield further insights for putative combination therapies targeting TP53-mutant GC.

We report the sex for our mouse tumor models (Table S3), but larger cohort sizes are required to confidentially investigate potential sex differences. Our human GC analysis is limited by the lack of sex and gender information for the patient specimen included in the tissue microarrays.

## STAR★Methods

### Key resources table

**Table:** 

REAGENT or RESOURCE	SOURCE	IDENTIFIER
**Antibodies**		

Anti-β-Actin antibody, mouse, clone AC-74	Merck	Cat# A2228; RRID:AB_476697
Anti-β-Catenin antibody, mouse, clone 14/Beta-Catenin (RUO)	BD Biosciences	Cat# 610153; RRID:AB_397554
Anti-GAPDH antibody, mouse monoclonal, clone GAPDH-71.1	Merck	Cat# G9545; RRID:AB_796208
Anti-IL6 antibody, clone MP5-20FS	BioXCell	Cat# BE0046; RRID:AB_ 1107709
Anti-mouse Immunoglobulins/HRP, polyclonal goat	Agilent	Cat# P0447; RRID:AB_ 2617137
Anti-p53 antibody, rabbit monoclonal, clone D2H9O)	Cell Signaling Technology	Cat# 32532; RRID:AB_2757821
Anti-phospho-STAT3 (Tyr705) antibody, rabbit	Cell Signaling Technology	Cat# 9131; RRID:AB_331586
Anti-Rabbit IgG antibody, biotinylated, goat	Vector Laboratories	Cat# BA-1000; RRID:AB_ 2313606
Ani-STAT3 antibody, rabbit monoclonal, clone 79D7	Cell Signaling Technology	Cat# 4904; RRID:AB_331269
IRDye 680RD Goat anti-Mouse IgG (H + L)	LI-COR	Cat# 926-68070; RRID:AB_10956588
IRDye 800CW Goat anti-Rabbit IgG (H + L)	LI-COR	Cat# 926-32211; RRID:AB_621843

**Biological samples**		

Tissue microarrays gastric adenocarcinoma	This paper	N/A
Tissue microarray gastro-esophageal junction adenocarcinoma	This paper	N/A
Tissue microarray normal mucosa	This paper	N/A

**Chemicals, peptides, and recombinant proteins**		

Advanced DMEM/F-12	Thermo Fisher Scientific	Cat# 12634010
Alt-R *Streptococcus pyogenes* Cas9 nuclease V3	Integrated DNA Technologies	Cat# 1081058
BBI608 (Stat3/Stemness inhibitor)	Sellekchem	Cat# 7977
cOmplete, Mini, EDTA-free Protease Inhibitor Cocktail	Roche	Cat# 11836170001
Cultrex Reduced Growth Factor Basement Membrane Extract, Type 2, Pathclear (RGF BME)	R&D Systems	Cat# 3533-005-02
DAB+ (3,3-Diaminobenzine), Liquid	Agilent	Cat# K346811-2
DPBS	Thermo Fisher Scientific	Cat# 14190144
Fetal Bovine Serum (FBS)	Bovogen biologicals	Cat# SFBS-AU
Gentle Cell Dissociation Reagent	STEMCELL Technologies	Cat# 100-0485
Intercept (TBS) Blocking Buffer	LI-COR	Cat# 927-60001
IntestiCult™ Organoid Growth Medium (Mouse)	STEMCELL Technologies	Cat# 06005
Lipofectamine RNAiMAX Transfection reagent	Thermo Fisher Scientific	Cat# 13778030
Mouse IL-4 Recombinant Protein, PeproTech®	Thermo Fisher Scientific	Cat# 214-14-20UG
MyTaq™ Red Mix	Meridian Bioscience	Cat# BIO-25044
Nuclease-Free Duplex Buffer	Integrated DNA Technologies	Cat# 11-01-03-01
NuPAGE 4 to 12%, Bis-Tris, 1.0–1.5 mm, Mini Protein Gels	Thermo Fisher Scientific	Cat# NP0335BOX
NuPAGE LDS Sample Buffer (4x)	Thermo Fisher Scientific	Cat# NP0007
NuPage MES SDS Running Buffer (20x)	Thermo Fisher Scientific	Cat# NP000202
NuPAGE Sample Reducing Agent (10x)	Thermo Fisher Scientific	Cat# NP0004
Opti-MEM	Thermo Fisher Scientific	Cat# 31985062
Penicillin/Streptomycin	Thermo Fisher Scientific	Cat# 15140122
PhosSTOP	Roche	Cat# 4906845001
Precision plus Protein Kaleidoscope Prestained Protein Standards	Bio-Rad Laboratories	Cat# 1610375
RIPA buffer	Merck	Cat# R0278
Skim Milk Powder	Devondale	N/A
Tamoxifen	Sigma-Aldrich	Cat# T5648
TRIzol™ Reagent	Thermo Fisher Scientific	Cat# 15596026
TrypLE Express	Thermo Fisher Scientific	Cat# 12604021

**Critical commercial assays**		

Applied Biosystems™ High-Capacity cDNA Reverse Transcription Kit	Thermo Fisher Scientific	Cat# 4368813
Pierce™ BCA Protein Assay Kit	Thermo Fisher Scientific	Cat# 23225
RealTime-GloTM MT Cell Viability Assay Kit	Promega Corporation	Cat# G9712
RNeasy Plus Micro Kit	Qiagen	Cat# 74034
SensiMix™ SYBR® Hi-ROX Kit	Meridian Bioscience	Cat# QT605-05
VECTASTAIN® Elite® ABC-HRP Kit, Peroxidase (Standard)	Vector Laboratories	Cat# PK-6100

**Deposited data**		

Supplementary Table Files MMC1 and MMC4	(Sanchez-Vega et al.)^27^	N/A
TP53 mutation data	COSMIC database: https://cancer.sanger.ac.uk/cosmic	N/A

**Experimental models: Cell lines**		

Mouse		N/A
*KPT* gastric cancer organoids	This paper	N/A
Stat3^KO^ organoids	This paper	N/A
TP53^CRISPR−KO/-^ organoids	This paper	N/A

**Experimental models: Organisms/strains**		

Mouse (all strains on C57BL/6 background):		N/A
Tg(Tff1-CreERT2)	(Thiem et al.)^28^	N/A
129S-*Trp53*^*tm2Tyj*^/J	The Jackson Laboratory	JAX 008652; RRID: IMSR_JAX:008652
*Apc*^*fl/*^*^fl^ (Apc580S)*	(Shibata et al.)^55^	N/A
B6.Cg-*Edil3*^*Tg(Sox2-cre)1Amc*^/J	The Jackson Laboratory	JAX 008454; RRID: IMSR_JAX:008454
B6.Cg-Tg(Pgk1-flpo)10Sykr/J	The Jackson Laboratory	JAX 011065; RRID: IMSR_JAX:011065
*IL6*^*−/−*^	(Kopf et al.)^56^	N/A
*IL11*^*−/−*^	This paper	N/A
*IL11ra*^*−/−*^	(Nandurkar et al.)^57^	N/A
*Kras*^*LSL-G12D*^	(Jackson et al.)^58^	N/A
*Pik3ca*^*LSL-H1047R*^	(Kinross et al.)^59^	N/A
*Pten*^*flox/-*^	(Suzuki et al.)^60^	N/A
*sgp130Fc*	(Rabe et al.)^61^	N/A

**Oligonucleotides**		

Alt-R CRISPR-Cas9 crRNA, 2nmol, ACGATCCGGGCAATTTCCAT (Stat3)	Integrated DNA Technologies	N/A
Alt-R CRISPR-Cas9 crRNA, 2nmol, CTTCCACCCGGATAAGATGC (Trp53)	Integrated DNA Technologies	N/A
Alt-R CRISPR-Cas9 tracrRNA-ATTO™ 550	Integrated DNA Technologies	Cat# 1075928
See Table S4 for primers for qRT-PCR		N/A
See Table S5 for primers for Trp53 status analysis		N/A

**Software and algorithms**		

Aperio ImageScope (version 12.4)	Leica Biosystems	RRID:SCR_020993
Aperio ImageScope Nuclear v9 algorithm (version 9.2)	Leica Biosystems	N/A
HALO (version 3.5)	Indica Labs	RRID:SCR_018350
HALO – Area Quantification Algorithm	Indica Labs	N/A
HALO – Random Forest Tissue Classifier	Indica Labs	N/A

**Other**		

KMplot website	KMplot.com	N/A
KRAS pathway activation signature	(Pek et al.)^62^	N/A
PI3K pathway activation signature	(Zhang et al.)^63^	N/A
Signaling pathway activation analysis	(Tan et al.)^6^	N/A
IL6/IL11/GP130 dependent STAT3 signaling activation signature	This paper	N/A

### Resource availability

#### Lead contact

Further information and requests for resources and reagents should be directed to and will be fulfilled by the lead contact, Moritz F. Eissmann (moritz.eissmann@onjcri.org.au).

#### Materials availability

Reagents generated within this study are available upon request.

#### Data and code availability

• All data reported in our paper will be shared upon request from the lead contact.
• This paper does not report original code.
• Any additional information required to reanalyze the data reported in this paper is available from the lead contact upon request.

### Experimental model and study participant details

#### Animals

Animal experiments were approved and conducted in accordance with all relevant ethical regulations for animal studies including the Australian code for the care and use of animals for scientific purposes. All animal studies were approved by the animal ethics committee of the Ludwig Institute for Cancer Research, the Walter and Eliza Hall Institute of Medical Research, La Trobe University and Austin Health.

Mice were co-housed under specific pathogen-free (SPF) conditions and age-and gender-matched littermates were used for experiments. Across all our animal experiments, we used 53% and 47% males, but no differences in tumor, carcinoma or metastasis incidence was found between female and male mice (Table S3). All mouse strains and compound mutants were maintained on C57BL/6J background. Mutant alleles, including those with lox-STOP-lox (LSL)-flanked exons, have been previously described: Tg(Tff1-CreERT2) (hereafter named *Tff1*^*CreERT2*^),^28^ *Kras*^*LSL-G12D*^,^58^ *Pik3ca*^*LSL-H1047R*^,^59^ *Pten*^*flox*^,^60^ *Trp53*^*LSL-R172H*^ mice, 129S-*Trp53*^*tm2Tyj*^/J (JAX stock #008652),^8^ *Apc*^*fl/fl*^,^55^ *IL6*^*−/−*^*,*^56^ *IL11ra*^*−/−*^*,*^57^ *sgp130*.^61^ Compound *Tff1*^*CreERT2*^;*Apc*^*fl/fl*^ mutant mice are referred to as *Apc*^*KO*^ mice. *Il11*^*−/−*^ mice were generated using the EUCOMM/KOMP vector PRPGS00164-A-H10 (containing *Frt*-flanked β-gal reporter and *neo* selection cassettes, as well as *loxP* sites flanking exons 2–5) for targeting of mouse G4 ES cells (C57BL/6Ncr x 129S6/SvEvTac) and subsequent selection with 200 μg/mL G418. Following screening by long-range PCR and Southern blot analysis, 2 clones were injected int C57BL/6J blastocysts. Resultant chimeras from ES cell clone 1E9 were mated with wild-type C57BL/6J mice to identify germline transmission of the mutant allele, prior to subsequent mating with *CAGGS-FlpO* and *Sox2-Cre* transgenic mice to subsequentially remove the *Frt*-flanked *neo* selection cassette and *loxP*-flanked *Il11* exons, respectively.

#### Organoids

Mouse gastric tumor organoids were established from the invasive front of intestinal adenocarcinoma bearing *KPT* mice and.^31^^,^^64^ After euthanasia of the mouse, the stomach was isolated, cut along the greater curvature and then washed twice with ice-cold PBS to remove stomach contents as well as mucus. Stomach tumors were dissected, washed with ice-cold PBS, and sliced into small pieces. Then, 20 mL of ambient Gentle Cell Dissociation Reagent was added to the tumor pieces and the mix was incubated for 20 min with agitation at room temperature. After letting the tissue pieces settle by gravity, the supernatant was discarded. The tissue pieces were resuspended with 10 mL ice-cold PBS and the tube was shaken vigorously for 20 s to release glands from underlying stromal tissue. Then, the pieces settled by gravity before transferring the supernatant to a new tube and centrifugation (1500 rpm, 4°C, 5 min). After resuspending the pellet 1 mL Advanced DMEM-F12 supplemented with Penicillin/Streptomycin (1:100 dilution), the suspension was strained through a 70 μm filter and centrifuged (1500 rpm, 4°C, 5 min). The supernatant was discarded before the pellet was resuspended in an appropriate amount of matrigel (RGF BME). Fifty μL of this cell-matrigel suspension were seeded in each well of a pre warmed 24-well tissue culture plate. The domes were allowed to set by incubation for 10 min at 37°C. Lastly, each well was supplemented with 500 μL of complete organoid medium and the plate incubated at 37°C with 10% CO_2_.

#### Human GC tissue microarray

The conducted research using patient samples was conducted in compliance with all relevant regulations. Collection and usage of human gastric cancer tissues was approved by the Austin Health ethics committee (HREC/15/Austin/359) with a waiver of consent.

Tissue microarrays (TMA) were prepared from gastric (*n* = 193), gastro-esophageal junction (*n* = 66) adenocarcinomas and normal mucosa (*n* = 80; from GC blocks near surgical resection margins, uninvolved by tumor) diagnosed at Austin Health between 2001 and 2014, for whom clinical, treatment and follow-up data had been retrospectively collected with human ethics approval. TMAs were produced from representative FFPE blocks of tumor, sampling 3 × 1 mm cores per patient. Pathology of each core of the TMA was confirmed post generation by a gastrointestinal pathologist (DW). Clinical and pathological characteristics of the patient cohort stratified for pSTAT3 staining positivity are summarized in Table S2. Sex and gender information of the patient cohort was not available.

### Method details

#### *In vivo* experiments

##### Cre/lox genetic modified models

Expression of the latent LSL mutant alleles was induced by intraperitoneal (IP) injection of tamoxifen (Sigma-Aldrich, Cat #T5648) in 10% Ethanol, 90% sunflower oil vehicle at 50 mg/kg body weight doses, twice daily for three consecutive days in 6–9-week-old mice that carry the *Tff1*^*CreERT2*^ allele. Upon tamoxifen administration mice were clinically monitored and euthanized at ethical or experimental endpoint (whichever occurred first). Sick mice that reached ethical endpoints 72 and 239 days post tamoxifen administration were euthanized and analyzed. Mice not showing signs of sickness were euthanized and analyzed between 252 and 280 days post tamoxifen administrations. Organs of interest were dissected and processes for histology and biochemical and molecular analysis.

##### Subcutaneous GC organoid allograft model

6–8-week-old mice were subcutaneously injected with 900 mechanically disrupted *KPT* GC organoids in 1:1 PBS and RGF BME (R&D Systems, Cat#3533-005-02) vehicle (equivalent of approximately 100,000 cells) into the right flank. An equal number of age-matched male and female host mice were used per experiment. Mice were monitored, and tumors were measured with a caliper (Mitutoyo Tools) three times per week. Tumor volumes were calculated with the formula: (length x (width)^2^)/2. For treatment experiments, drug administration commenced when tumors reached ∼100 mm^3^ BBI608 (Stat3/Stemness inhibitor,^30^ Sellekchem, Cat #S7977) was administered every three days at 20 mg/kg doses in vehicle (5% DMSO, 40% PEG300, 5% Tween80) and anti-IL6 antibody (clone MP5-20FS, BioXCell) was IP injected every three days at 10 mg/kg in PBS vehicle. At experimental endpoint, tumors were dissected and weighed (tumor mass at endpoint). We observed that up to 20% of organoids gave rise to tumors with cystic, liquid-filled cores. Thus, once excised, these tumors were cut in half to drain the fluid prior to weighing. As longitudinal caliper measurements of tumor volumes may overestimate the tumor burden of fluid-filled lesions, we also determine tumor burden by weight at the end of the experiments.

##### Tissue collection

Stomachs, liver, lungs and other organs of interest (for Cre/lox models) and tumors and adjacent tissues (for subcutaneous models) were resected, weighed and then tissue aliquots were snap-frozen for later RNA or protein isolation. Tumor aliquots from the invasive front within the submucosal layers were used to generate GC organoid cultures. The remaining tumor aliquots, tissues and organs were fixed in 10% neutral buffer formalin and embedded in paraffin blocks for subsequent histological and pathological analysis.

##### Histological and pathological analysis

Hematoxylin-Eosin staining of formalin-fixed paraffin-embedded tissues was performed according to “Theory and practice of histological techniques”.^65^

Pathological assessment was performed by a gastrointestinal pathologist (DW). *KP*, *KPP* and *KPT* mouse tumors were classified using both WHO and Lauren classifications,^66^ and histopathologically assessed in accordance with AJCC cancer staging manual.^67^ Histopathological assessment of *KPT* mouse tumors is summarized in Table S1. For the Kaplan-Meier survival analysis (Figure 1D), *KPT* mice were removed from the analysis when they displayed sarcomas in non-stomach organs or lymphomas in the thymus (*n* = 3) or when they reached ethical endpoint without bearing stomach tumors (*n* = 4). Osteosarcomas, lymphomas in the thymus and other anatomical sites arise from loss of wild-type TP53 expression in non-stomach body cells,^68^ here through the presence of *Trp53*^*LoE*^/non-Cre-recombined *Trp53*^*LSL−172H*^ body cells.

##### CRISPR-Cas9 knockout organoid generation

Stat3^KO^ and Trp53^CRISPR−KO/-^ organoids were established using Alt-R CRISPR-Cas9 system (Integrated DNA Technologies), with crisprRNA ACGATCCGGGCAATTTCCAT (Stat3), CTTCCACCCGGATAAGATGC (Trp53) and ATTO550-labeled tracrRNA.^69^ Each crRNA was mixed with the fluorescently labeled (ATTO 550) tracrRNA in equimolar concentrations to create the guide RNA (gRNA) at a final concentration of 1 μM. After heating the mix at 95°C for 5 min, it was allowed to cool down to room temperature. The ribonucleoprotein (RNP) complex was assembled by combining 3 μL of 1 μM gRNA, 3 μL of 1 μM Cas9 nuclease and 44 μL of Opti-MEM medium (per well of 48-well plate) and incubating at room temperature for 5 min. To form the transfection complexes, the RNP complex was mixed with 2.4 μL Lipofectamine RNAiMAX transfection reagent and 47.6 μL Opti-MEM medium (per well of 48-well plate) and incubated at room temperature for 20 min. Meanwhile, the organoids were dissociated into a single-cell suspension and 200 μL of this organoid cell suspension were placed into one well of a 48-well tissue culture plate. Then, 100 μL of transfection complexes were added to each well and incubated at 37°C with 10% CO_2_. After approximately 24 h, the organoid cells were centrifuged (1500 rpm, 4°C, 5 min) and the pellet resuspended in 500 μL FACS buffer. The suspension was passed through a 70 μm cell strainer before ATTO 550-positive single live cells were sorted into a 1.5 mL tube containing complete organoid medium. The sorted ATTO 550-positive organoid cells were centrifuged (1500 rpm, 4°C, 5 min) and the supernatant discarded before the pellet was resuspended in RGF BME. For one dome, 50 μL of the suspension were seeded in a well of a pre warmed 24-well tissue culture plate. The dome was allowed to set by incubating he plate at 37°C for 10 min. Lastly, 500 μL of complete organoid medium (+1:100 Penicillin/Streptomycin) were added to each well and the plate incubated at 37°C with 10% CO_2_. In general, re-grow of organoids from sorted cells of dissociated organoids can be seen after a few days and organoids recover their typical morphology and size 7 to 10 days after FACS. Media was exchanged every 5 days. Single organoids were handpicked under the microdissection microscope to establish clonal cultures. Successful knockout was confirmed at the proteomic level by western blotting.

##### Organoid *in vitro* growth assay

The RealTime-Glo MT Cell Viability Assay Kit (Promega, Cat# G9712) was used to assess organoid *in vitro* viability and proliferation (adapted from Morrow et al.^70^). Organoids were seeded at a concentration of 10 organoids in 35μL of RGF BME in a 96-well tissue culture plate placed on ice. After allowing the RGF BME to solidify for 10 min at 37°C, 100 μL of organoid medium (1% Penicillin/Streptomycin) containing 1X MT Cell Viability Substrate and 1X NanoLuc Enzyme were added. Following 24h of incubation at 37°C with 10% CO_2_, luminescence was measured. Each well was supplemented with another 100μL of organoid medium (1% Penicillin/Streptomycin) containing 1X MT Cell Viability Substrate and 1X NanoLuc Enzyme before incubation at 37°C with 10% CO_2_. Luminescence was measured every 24h for 9 days. Experiments were performed in three independent biological replicates consisting of technical quadruplicates.

##### Organoid- BMDM co-culture experiment

Bone-marrow derived macrophages (BMDM) were established from C57BL/6 wild-type mice.^31^ Bone marrow was flushed from the femur and tibia with sterile PBS. After bone marrow cells were washed twice with PBS, they were filtered through a 100 mm sieve. Filtered cell suspensions were cultured in DMEM containing 10% (v/v) FCS and L929 conditioned medium for 7 days. Media was changed every second day until fully differentiated into BMDM. 1.5 × 10^5^ day 6 BMDM cells were mixed and seeded together with 300 dissociated GC organoids in 100 μL RGF BME domes in 24-well plate wells and incubated for 72 h in 100 μL IntestiCult organoid growth medium (STEMCELL Technologies) containing 20% L929 conditioned medium and 20 ng/mL IL4. At endpoint, growth media were removed, and lysis buffer added to assay wells followed by subsequent RNA isolation and qRT-PCR expression analysis.

##### RNA extraction and qRT-PCR analysis

Total RNA from snap frozen tissue was extracted using TrizolReagent (Life Technologies, Cat# 15596026), while RNA from organoids was extracted using the RNeasy Plus Micro Kit (QIAGEN, Cat# 74034). cDNA was prepared from 2 μg RNA using the High-capacity cDNA Reverse Transcription kit (Applied Biosystems, Cat# 4368813) according to the manufacturer’s protocol.

Quantitative RT-PCR analyses were performed in technical triplicates with SensiMix SYBR kit (Bioline, Cat# QT605–20) using the ViiA 7 Real-Time PCR System (Life Technologies). Primer sequences used are in Table S4.

##### Immunohistochemical analysis

Paraffin-embedded formalin fixed 4 μm tissue section on charged microscopy glass slides were dewaxed and rehydrated. Antigen retrieval was performed in citrate buffer in a microwave pressure cooker (pH 6 for 15 min). Sections were blocked in 10% (v/v) normal goat serum for 1 h at 20°C–25°C in a humidified chamber, incubated overnight at 4°C with primary antibodies and for 1h at room temperature with secondary antibodies. Visualisation was achieved using 3,3-Diaminobenzine (DAB, DAKO). Primary antibodies used were: anti-pSTAT3 at 1.33 μg/mL (Tyr705, Cell Signaling, Cat # 9131), anti-β-catenin at 1.25 μg/mL (BD Biosciences, Cat # 610153) and secondary antibodies were: anti-rabbit-biotinylated at 0.75 μg/mL (Vector labs, BA-100, in conjunction with VECTASTAIN ABC kit), anti-mouse-HRP at 5 μg/mL (Dako, Cat #P0447).

For quantification of tissue sections, sections were scanned (Aperio AT2 Leica Scanner) and analyzed with Image Scope software (Leica Biosystems, version 12.4). For mouse tumor tissues, nuclear pSTAT3 positive cells were quantified with Image Scope’s Nuclear v9 algorithm (version 9.2) at default settings and normalized against the total area assessed.

For the human GC TMAs, HALO software (Indica Labs, version 3.5) was used to distinguish tumor/epithelial and stromal cell compartments (Random Forest Tissue Classifier), as well as to quantify positive staining (Area Quantification Algorithm). Tissue cores that had less than 20% tumor or epithelial cells were removed from tumor/epithelial compartment analysis. Cores with strong non-specific background pSTAT3 staining (predominantly in gastric gland lumen) or cores with greater 40% tissue loss were excluded from the analysis.

##### Protein extraction and immunoblot analysis

Protein lysates from snap-frozen tissue were prepared using the TissueLyser II (Qiagen) and RIPA lysis buffer (Sigma). Cultured organoids were directly lysed by adding RIPA lysis buffer to the organoid domes and mechanical disruption by 20-time pipetting. Protein concentration was measured using the Pierce BCA Protein Assay (Thermo Fisher) according to the manufacturer’s instructions. For Immunoblotting, 25–50 μg of protein were mixed with 1X NuPAGE LDS Sample Buffer and 1X NuPAGE Sample Reducing Agent (both Thermo Fisher). After denaturation of samples at 90°C for 10 min, samples as well as 10 μL Precision Plus Protein Kaleidoscope Prestained Protein Standard (BIO-RAD) were loaded onto a precast 4–12% Bis-Tris polyacrylamide NuPAGE protein gel (Thermo Fisher) and run in the Novex mini-cell running tank (Invitrogen) with 1X NuPAGE MES SDS Running Buffer (Thermo Fisher) at 120 V for approximately 1 h. After separated proteins were transferred onto a PVDF membrane (Millipore) using the iBLOT dry blotting system (Invitrogen), membranes were blocked with 5% skim milk powder in TBST at room temperature for at least 1 h. Then, membranes were incubated with primary antibody overnight at 4°C. The membranes were washed three times with TBST, followed by an incubation with the secondary fluorescent-labelled antibody at room temperature for.1 h. Immunoblots were imaged using the Odyssey CLx Infrared Imaging System (LI-COR). Antibodies used were: primary: pSTAT3 (Tyr705, Cell Signaling #9131), STAT3 (Cell Signaling Technology #4904), TP53 (D2H90, Cell Signaling Technology, #32532), beta-ACTIN (AC-74, Merck #A2228) and GAPDH (Merck #G9545); secondary antibodies: fluorescent-conjugated (LI-COR Biosciences #926–68071).

##### *Trp53* status analysis

From 13 of 19 tumor-bearing KPT mice genomic DNA from the endogenous KPT tumor, or tumor-derived organoids were analyzed for their Trp53 allele status using two independent PCR reactions. For the remaining 6 tumor-bearing KPT mice, no fresh frozen tissue was collected and therefore the Trp53 allele status could not be determined. PCR A, adapted from Olive et al.,^8^ enables detection of the GoF allele (330 bp band) and the WT allele (290 bp band). PCR B allows for detection of the LoF allele (370 bp band) but does not distinguish between the WT and GoF allele (174 bp bands). For both PCRs, 100–150 ng of genomic DNA sample was used with PCR cycling conditions: 95°C for 5 min, 35 cycles of 95°C for 30 s, 55°C (PCR A) or 53°C (PCR B) for 30 s, and 72°C for 30 s, final elongation step 72°C for 5 min. Respective oligonucleotide primers (Table S5) were used at a concentration of 500 nM in 2× PCR premix reagent (My Taq Red PCR Mix, Catalog #: MER-BIO-25044, Bioline).

##### Kaplan-Meier survival analysis

All Kaplan–Meier survival analysis were performed with KMplot (KMplot.com).^71^ RNA-seq data from stomach adenocarcinoma (*N* = 375) patients within the pan-cancer dataset were analyzed. All other settings were kept as default. For stratification into high and low expression either the “auto select best cutoff” option or median expression was selected, as indicated in the corresponding figure legends. The best cutoff algorithm calculates all possible cutoff values between the lower and upper quartiles and then selects the best performing threshold.^72^ A False Discovery Rate (FDR) is provided in the figure legends were applicable. For the STAT3 gene signature the “Use Multiple GENES” feature and “mean expression of selected genes” option were used to generate the Kaplan-Meier survival analysis graph.

#### Bioinformatic analysis

##### Signaling pathway alteration analysis

Sanchez-Vega et al. have analyzed all tumor types from the TCGA dataset for frequency of signaling pathway alterations on a pathway level rather than individual gene mutations. Here, we have removed the esophageal tumor data from Sanchez-Vega et al.’s Supplementary Table Files MMC1 and MMC4.^27^ The resulting stomach adenocarcinoma selective dataset was interrogated, and top alternated pathways are shown in Figure S1A.

##### Signaling pathway activation analysis

Signaling pathway activation analysis is based on RNA sequencing expression analysis of gene expression signatures. KRAS and PI3K pathway activation gene signatures were used as defined by Pek et al.^62^ and Zhang et al.^63^ TP53 pathway activation was not analyzed but TP53 genetic alteration frequencies are presented. Definition of a TP53 pathway activation gene signature is difficult, due to different gene signatures being associated with loss-of-function versus gain-of-function TP53 mutations as well as non-transcriptional activity of mutant TP53. TP53 mutation data were downloaded from the COSMIC database.

Signaling pathway activation analysis was adapted from Tan et al*.*^6^ Briefly, level 3 TCGA RNA-seq normalized data for 415 gastric cancer samples and 35 normal gastric samples, and their corresponding clinical information, were downloaded from the Broad Institute TCGA Genome Data Analysis Center Firehose. Gene expression values were log-transformed and centered to the standard deviation of the median across the samples included in the analysis. A μ score was calculated for each sample by averaging the standardized expression values of pathway signature genes. A pathway is deemed activated in a tumor sample if its μ score in that sample surpasses the 90th quantile of μ scores calculated across all normal samples.

##### IL6/Il11/GP130/STAT3 activation gene signature

STAT3 signaling can be induced by different stimuli and may result in activation of gene expression in different target gene subsets in a context dependent manner. Here we defined an IL6 and IL11 cytokine and GP130 dependent STAT3 signaling activation gene signature for gastric cancer. Previously we stimulated *Gp130 F/F* mutant mice with either recombinant IL6 and IL11 and performed RNA sequencing and STAT3-Chip/Seq analysis.^31^ Here, we selected the top 12 upregulated genes from the IL11 stimulation and retained all genes which are equally upregulated in IL6 stimulated tumors and where STAT3 did bind to the gene body or promoter in the STAT3-ChipSeq analysis. Two genes were removed due to lack of human homologue genes. Four genes were removed due extensive non-tumor cell expression based on mouse stomach and *gp130 F/F* tumor single cell RNA sequencing data^73^ as well as human protein atlas expression distribution.^74^ One gene was removed due to higher expression in normal tissue than STAD, resulting in a final five gene signature: *SOCS3*, *PIM3*, *OSMR*, *CLDN12* and *STAT3*.

### Quantification and statistical analysis

All experiments were conducted at least twice, if not otherwise indicated and for animal experiments with ≥3 sex-and aged-matched mice per group. For drug treatment experiments animals were randomized into treatment groups. Tumor growth measurements were performed blinded to treatment or genetic cohort conditions. No data was excluded from the analysis, if not indicated otherwise. Data used to generate the figure is provided in a Source File. GraphPad Prism 9 software was used to calculate means, standard error of the mean and was used to perform statistical testing. For two group comparisons, unpaired two-sided Student’s *t*-test was performed, either with or without Welch correction depending on deviation F of the data. If not normally distributed, Mann Whitney test was performed. Data comparison of more than two groups was done with one-way ANOVA, with multiple comparison testing by Tukey (when comparing the mean of each column with the mean of every other column), Dunnett (when comparing each column mean with the mean of a control column) or Sidak (when comparing the means of preselected pairs of columns). Kruskal-Wallis with Dunn’s multiple comparison testing was used for non-normally distributed datasets. Kaplan-Meier survival analysis Hazard ratios and *p* values were calculated with Mantel-Cox’s Log rank test and contingency analyses was done using two-tailed Fisher’s exact test.

## Supplemental information

Document S1. Figures S1–S7 and Tables S1–S5

Document S2. Article plus supplemental information

## References

[1] Sung H., Ferlay J., Siegel R.L., Laversanne M., Soerjomataram I., Jemal A., Bray F. (2021). Global Cancer Statistics 2020: GLOBOCAN Estimates of Incidence and Mortality Worldwide for 36 Cancers in 185 Countries. CA A Cancer J. Clin..

[2] Arnold M., Rutherford M., Lam F., Bray F., Ervik M., Soerjomataram I. (2019). https://gco.iarc.fr/survival/survmark.

[3] Cancer Genome Atlas Research Network (2014). Comprehensive molecular characterization of gastric adenocarcinoma. Nature.

[4] Cristescu R., Lee J., Nebozhyn M., Kim K.M., Ting J.C., Wong S.S., Liu J., Yue Y.G., Wang J., Yu K. (2015). Molecular analysis of gastric cancer identifies subtypes associated with distinct clinical outcomes. Nat. Med..

[5] Wang K., Yuen S.T., Xu J., Lee S.P., Yan H.H.N., Shi S.T., Siu H.C., Deng S., Chu K.M., Law S. (2014). Whole-genome sequencing and comprehensive molecular profiling identify new driver mutations in gastric cancer. Nat. Genet..

[6] Tan S.H., Swathi Y., Tan S., Goh J., Seishima R., Murakami K., Oshima M., Tsuji T., Phuah P., Tan L.T. (2020). AQP5 enriches for stem cells and cancer origins in the distal stomach. Nature.

[7] Lang G.A., Iwakuma T., Suh Y.A., Liu G., Rao V.A., Parant J.M., Valentin-Vega Y.A., Terzian T., Caldwell L.C., Strong L.C. (2004). Gain of function of a p53 hot spot mutation in a mouse model of Li-Fraumeni syndrome. Cell.

[8] Olive K.P., Tuveson D.A., Ruhe Z.C., Yin B., Willis N.A., Bronson R.T., Crowley D., Jacks T. (2004). Mutant p53 gain of function in two mouse models of Li-Fraumeni syndrome. Cell.

[9] Aschauer L., Muller P.A.J. (2016). Novel targets and interaction partners of mutant p53 Gain-Of-Function. Biochem. Soc. Trans..

[10] Liu Y., Chen C., Xu Z., Scuoppo C., Rillahan C.D., Gao J., Spitzer B., Bosbach B., Kastenhuber E.R., Baslan T. (2016). Deletions linked to TP53 loss drive cancer through p53-independent mechanisms. Nature.

[11] Sabapathy K., Lane D.P. (2018). Therapeutic targeting of p53: all mutants are equal, but some mutants are more equal than others. Nat. Rev. Clin. Oncol..

[12] Yu X., Vazquez A., Levine A.J., Carpizo D.R. (2012). Allele-specific p53 mutant reactivation. Cancer Cell.

[13] Liu D.S.H., Read M., Cullinane C., Azar W.J., Fennell C.M., Montgomery K.G., Haupt S., Haupt Y., Wiman K.G., Duong C.P. (2015). APR-246 potently inhibits tumour growth and overcomes chemoresistance in preclinical models of oesophageal adenocarcinoma. Gut.

[14] Santhanam U., Ray A., Sehgal P.B. (1991). Repression of the interleukin 6 gene promoter by p53 and the retinoblastoma susceptibility gene product. Proc. Natl. Acad. Sci. USA.

[15] Margulies L., Sehgal P.B. (1993). Modulation of the human interleukin-6 promoter (IL-6) and transcription factor C/EBP beta (NF-IL6) activity by p53 species. J. Biol. Chem..

[16] Nowak D.G., Cho H., Herzka T., Watrud K., DeMarco D.V., Wang V.M.Y., Senturk S., Fellmann C., Ding D., Beinortas T. (2015). MYC Drives Pten/Trp53-Deficient Proliferation and Metastasis due to IL6 Secretion and AKT Suppression via PHLPP2. Cancer Discov..

[17] Wormann S.M., Song L., Ai J., Diakopoulos K.N., Kurkowski M.U., Gorgulu K., Ruess D., Campbell A., Doglioni C., Jodrell D. (2016). Loss of P53 Function Activates JAK2-STAT3 Signaling to Promote Pancreatic Tumor Growth, Stroma Modification, and Gemcitabine Resistance in Mice and Is Associated With Patient Survival. Gastroenterology.

[18] Schulz-Heddergott R., Stark N., Edmunds S.J., Li J., Conradi L.C., Bohnenberger H., Ceteci F., Greten F.R., Dobbelstein M., Moll U.M. (2018). Therapeutic Ablation of Gain-of-Function Mutant p53 in Colorectal Cancer Inhibits Stat3-Mediated Tumor Growth and Invasion. Cancer Cell.

[19] Fox J.G., Wang T.C. (2007). Inflammation, atrophy, and gastric cancer. J. Clin. Invest..

[20] Tebbutt N.C., Giraud A.S., Inglese M., Jenkins B., Waring P., Clay F.J., Malki S., Alderman B.M., Grail D., Hollande F. (2002). Reciprocal regulation of gastrointestinal homeostasis by SHP2 and STAT-mediated trefoil gene activation in gp130 mutant mice. Nat. Med..

[21] Putoczki T.L., Thiem S., Loving A., Busuttil R.A., Wilson N.J., Ziegler P.K., Nguyen P.M., Preaudet A., Farid R., Edwards K.M. (2013). Interleukin-11 is the dominant IL-6 family cytokine during gastrointestinal tumorigenesis and can be targeted therapeutically. Cancer Cell.

[22] Huynh J., Etemadi N., Hollande F., Ernst M., Buchert M. (2017). The JAK/STAT3 axis: A comprehensive drug target for solid malignancies. Semin. Cancer Biol..

[23] Huynh J., Chand A., Gough D., Ernst M. (2019). Therapeutically exploiting STAT3 activity in cancer - using tissue repair as a road map. Nat. Rev. Cancer.

[24] Jing B., Wang T., Sun B., Xu J., Xu D., Liao Y., Song H., Guo W., Li K., Hu M. (2020). IL6/STAT3 Signaling Orchestrates Premetastatic Niche Formation and Immunosuppressive Traits in Lung. Cancer Res..

[25] Huynh J., Baloyan D., Chisanga D., Shi W., O'Brien M., Afshar-Sterle S., Alorro M., Pang L., Williams D.S., Parslow A.C. (2021). Host IL11 Signaling Suppresses CD4(+) T cell-Mediated Antitumor Responses to Colon Cancer in Mice. Cancer Immunol. Res..

[26] Deng J.Y., Sun D., Liu X.Y., Pan Y., Liang H. (2010). STAT-3 correlates with lymph node metastasis and cell survival in gastric cancer. World J. Gastroenterol..

[27] Sanchez-Vega F., Mina M., Armenia J., Chatila W.K., Luna A., La K.C., Dimitriadoy S., Liu D.L., Kantheti H.S., Saghafinia S. (2018). Oncogenic Signaling Pathways in The Cancer Genome Atlas. Cell.

[28] Thiem S., Eissmann M.F., Elzer J., Jonas A., Putoczki T.L., Poh A., Nguyen P., Preaudet A., Flanagan D., Vincan E. (2016). Stomach-Specific Activation of Oncogenic KRAS and STAT3-Dependent Inflammation Cooperatively Promote Gastric Tumorigenesis in a Preclinical Model. Cancer Res..

[29] Phesse T.J., Buchert M., Stuart E., Flanagan D.J., Faux M., Afshar-Sterle S., Walker F., Zhang H.H., Nowell C.J., Jorissen R. (2014). Partial inhibition of gp130-Jak-Stat3 signaling prevents Wnt-beta-catenin-mediated intestinal tumor growth and regeneration. Sci. Signal..

[30] Li Y., Rogoff H.A., Keates S., Gao Y., Murikipudi S., Mikule K., Leggett D., Li W., Pardee A.B., Li C.J. (2015). Suppression of cancer relapse and metastasis by inhibiting cancer stemness. Proc. Natl. Acad. Sci. USA.

[31] Eissmann M.F., Dijkstra C., Jarnicki A., Phesse T., Brunnberg J., Poh A.R., Etemadi N., Tsantikos E., Thiem S., Huntington N.D. (2019). IL-33-mediated mast cell activation promotes gastric cancer through macrophage mobilization. Nat. Commun..

[32] Ikari N., Serizawa A., Mitani S., Yamamoto M., Furukawa T. (2019). Near-Comprehensive Resequencing of Cancer-Associated Genes in Surgically Resected Metastatic Liver Tumors of Gastric Cancer. Am. J. Pathol..

[33] Nemtsova M.V., Kalinkin A.I., Kuznetsova E.B., Bure I.V., Alekseeva E.A., Bykov I.I., Khorobrykh T.V., Mikhaylenko D.S., Tanas A.S., Kutsev S.I. (2020). Clinical relevance of somatic mutations in main driver genes detected in gastric cancer patients by next-generation DNA sequencing. Sci. Rep..

[34] Parikh N., Hilsenbeck S., Creighton C.J., Dayaram T., Shuck R., Shinbrot E., Xi L., Gibbs R.A., Wheeler D.A., Donehower L.A. (2014). Effects of TP53 mutational status on gene expression patterns across 10 human cancer types. J. Pathol..

[35] Wang Z., Burigotto M., Ghetti S., Vaillant F., Tan T., Capaldo B.D., Palmieri M., Hirokawa Y., Tai L., Simpson D.S. (2024). Loss-of-function but not gain-of-function properties of mutant TP53 are critical for the proliferation, survival and metastasis of a broad range of cancer cells. Cancer Discov..

[36] Lim B.H., Soong R., Grieu F., Robbins P.D., House A.K., Iacopetta B.J. (1996). p53 accumulation and mutation are prognostic indicators of poor survival in human gastric carcinoma. Int. J. Cancer.

[37] Ott K., Vogelsang H., Mueller J., Becker K., Müller M., Fink U., Siewert J.R., Höfler H., Keller G. (2003). Chromosomal instability rather than p53 mutation is associated with response to neoadjuvant cisplatin-based chemotherapy in gastric carcinoma. Clin. Cancer Res..

[38] Mrozek A., Petrowsky H., Sturm I., Kraus J., Hermann S., Hauptmann S., Lorenz M., Dorken B., Daniel P.T. (2003). Combined p53/Bax mutation results in extremely poor prognosis in gastric carcinoma with low microsatellite instability. Cell Death Differ..

[39] Park S., Lee J., Kim Y.H., Park J., Shin J.W., Nam S. (2016). Clinical Relevance and Molecular Phenotypes in Gastric Cancer, of TP53 Mutations and Gene Expressions, in Combination With Other Gene Mutations. Sci. Rep..

[40] Pallocca M., Goeman F., De Nicola F., Melucci E., Sperati F., Terrenato I., Pizzuti L., Casini B., Gallo E., Amoreo C.A. (2018). Coexisting YAP expression and TP53 missense mutations delineates a molecular scenario unexpectedly associated with better survival outcomes in advanced gastric cancer. J. Transl. Med..

[41] Tahara T., Tahara S., Horiguchi N., Okubo M., Terada T., Yamada H., Yoshida D., Omori T., Osaki H., Maeda K. (2019). Molecular subtyping of gastric cancer combining genetic and epigenetic anomalies provides distinct clinicopathological features and prognostic impacts. Hum. Mutat..

[42] Migliavacca M., Ottini L., Bazan V., Agnese V., Corsale S., Macaluso M., Lupi R., Dardanoni G., Valerio M.R., Pantuso G. (2004). TP53 in gastric cancer: mutations in the l3 loop and LSH motif DNA-binding domains of TP53 predict poor outcome. J. Cell. Physiol..

[43] Tahara T., Shibata T., Okamoto Y., Yamazaki J., Kawamura T., Horiguchi N., Okubo M., Nakano N., Ishizuka T., Nagasaka M. (2016). Mutation spectrum of TP53 gene predicts clinicopathological features and survival of gastric cancer. Oncotarget.

[44] ICGC/TCGA Pan-Cancer Analysis of Whole Genomes Consortium (2020). Pan-cancer analysis of whole genomes. Nature.

[45] Baslan T., Morris J.P., Zhao Z., Reyes J., Ho Y.J., Tsanov K.M., Bermeo J., Tian S., Zhang S., Askan G. (2022). Ordered and deterministic cancer genome evolution after p53 loss. Nature.

[46] Rose-John S., Jenkins B.J., Garbers C., Moll J.M., Scheller J. (2023). Targeting IL-6 trans-signalling: past, present and future prospects. Nat. Rev. Immunol..

[47] Shin S.Y., Choi C., Lee H.G., Lim Y., Lee Y.H. (2012). Transcriptional regulation of the interleukin-11 gene by oncogenic Ras. Carcinogenesis.

[48] Ancrile B., Lim K.H., Counter C.M. (2007). Oncogenic Ras-induced secretion of IL6 is required for tumorigenesis. Genes Dev..

[49] Sparmann A., Bar-Sagi D. (2004). Ras-induced interleukin-8 expression plays a critical role in tumor growth and angiogenesis. Cancer Cell.

[50] Bao Y., Wu Y., Tao B., Sun R., Lin T., Zheng Y., Zhu X., Shen H., Chen W., Fan Y. (2020). Super-enhancers modulate interleukin-6 expression and function in cancers. Transl. Cancer Res..

[51] Efe G., Dunbar K.J., Sugiura K., Cunningham K., Carcamo S., Karaiskos S., Tang Q., Cruz-Acuña R., Resnick-Silverman L., Peura J. (2023). p53 gain-of-function mutation induces metastasis via Brd4-dependent Csf-1 expression. Cancer Discov..

[52] Angevin E., Tabernero J., Elez E., Cohen S.J., Bahleda R., van Laethem J.L., Ottensmeier C., Lopez-Martin J.A., Clive S., Joly F. (2014). A phase I/II, multiple-dose, dose-escalation study of siltuximab, an anti-interleukin-6 monoclonal antibody, in patients with advanced solid tumors. Clin. Cancer Res..

[53] Dorff T.B., Goldman B., Pinski J.K., Mack P.C., Lara P.N., Van Veldhuizen P.J., Quinn D.I., Vogelzang N.J., Thompson I.M., Hussain M.H.A. (2010). Clinical and correlative results of SWOG S0354: a phase II trial of CNTO328 (siltuximab), a monoclonal antibody against interleukin-6, in chemotherapy-pretreated patients with castration-resistant prostate cancer. Clin. Cancer Res..

[54] Huseni M.A., Wang L., Klementowicz J.E., Yuen K., Breart B., Orr C., Liu L.F., Li Y., Gupta V., Li C. (2023). CD8(+) T cell-intrinsic IL-6 signaling promotes resistance to anti-PD-L1 immunotherapy. Cell Rep. Med..

[55] Shibata H., Toyama K., Shioya H., Ito M., Hirota M., Hasegawa S., Matsumoto H., Takano H., Akiyama T., Toyoshima K. (1997). Rapid colorectal adenoma formation initiated by conditional targeting of the Apc gene. Science.

[56] Kopf M., Baumann H., Freer G., Freudenberg M., Lamers M., Kishimoto T., Zinkernagel R., Bluethmann H., Köhler G. (1994). Impaired immune and acute-phase responses in interleukin-6-deficient mice. Nature.

[57] Nandurkar H.H., Robb L., Tarlinton D., Barnett L., Köntgen F., Begley C.G. (1997). Adult mice with targeted mutation of the interleukin-11 receptor (IL11Ra) display normal hematopoiesis. Blood.

[58] Jackson E.L., Willis N., Mercer K., Bronson R.T., Crowley D., Montoya R., Jacks T., Tuveson D.A. (2001). Analysis of lung tumor initiation and progression using conditional expression of oncogenic K-ras. Genes Dev..

[59] Kinross K.M., Montgomery K.G., Kleinschmidt M., Waring P., Ivetac I., Tikoo A., Saad M., Hare L., Roh V., Mantamadiotis T. (2012). An activating Pik3ca mutation coupled with Pten loss is sufficient to initiate ovarian tumorigenesis in mice. J. Clin. Invest..

[60] Suzuki A., Yamaguchi M.T., Ohteki T., Sasaki T., Kaisho T., Kimura Y., Yoshida R., Wakeham A., Higuchi T., Fukumoto M. (2001). T cell-specific loss of Pten leads to defects in central and peripheral tolerance. Immunity.

[61] Rabe B., Chalaris A., May U., Waetzig G.H., Seegert D., Williams A.S., Jones S.A., Rose-John S., Scheller J. (2008). Transgenic blockade of interleukin 6 transsignaling abrogates inflammation. Blood.

[62] Pek M., Yatim S.M.J.M., Chen Y., Li J., Gong M., Jiang X., Zhang F., Zheng J., Wu X., Yu Q. (2017). Oncogenic KRAS-associated gene signature defines co-targeting of CDK4/6 and MEK as a viable therapeutic strategy in colorectal cancer. Oncogene.

[63] Zhang Y., Kwok-Shing Ng P., Kucherlapati M., Chen F., Liu Y., Tsang Y.H., de Velasco G., Jeong K.J., Akbani R., Hadjipanayis A. (2017). A Pan-Cancer Proteogenomic Atlas of PI3K/AKT/mTOR Pathway Alterations. Cancer Cell.

[64] Flanagan D.J., Schwab R.H.M., Tran B.M., Phesse T.J., Vincan E. (2019). Isolation and Culture of Adult Intestinal, Gastric, and Liver Organoids for Cre-recombinase-Mediated Gene Deletion. Methods Mol. Biol..

[65] Bancroft J.D., Stevens A. (1990).

[66] Nagtegaal I.D., Odze R.D., Klimstra D., Paradis V., Rugge M., Schirmacher P., Washington K.M., Carneiro F., Cree I.A., WHO Classification of Tumours Editorial Board (2020). The 2019 WHO classification of tumours of the digestive system. Histopathology.

[67] Amin M.B., Greene F.L., Edge S.B., Compton C.C., Gershenwald J.E., Brookland R.K., Meyer L., Gress D.M., Byrd D.R., Winchester D.P. (2017). The Eighth Edition AJCC Cancer Staging Manual: Continuing to build a bridge from a population-based to a more "personalized" approach to cancer staging. CA A Cancer J. Clin..

[68] Donehower L.A., Harvey M., Slagle B.L., McArthur M.J., Montgomery C.A., Butel J.S., Bradley A. (1992). Mice deficient for p53 are developmentally normal but susceptible to spontaneous tumours. Nature.

[69] Huber A., Dijkstra C., Ernst M., Eissmann M.F. (2023). Generation of gene-of-interest knockouts in murine organoids using CRISPR-Cas9. STAR Protoc..

[70] Morrow R.J., Ernst M., Poh A.R. (2023). Longitudinal quantification of mouse gastric tumor organoid viability and growth using luminescence and microscopy. STAR Protoc..

[71] Nagy Á., Munkácsy G., Győrffy B. (2021). Pancancer survival analysis of cancer hallmark genes. Sci. Rep..

[72] Lanczky A., Gyorffy B. (2021). Web-Based Survival Analysis Tool Tailored for Medical Research (KMplot): Development and Implementation. J. Med. Internet Res..

[73] O'Keefe R.N., Carli A.L.E., Baloyan D., Chisanga D., Shi W., Afshar-Sterle S., Eissmann M.F., Poh A.R., Pal B., Seillet C. (2023). A tuft cell - ILC2 signaling circuit provides therapeutic targets to inhibit gastric metaplasia and tumor development. Nat. Commun..

[74] Karlsson M., Zhang C., Mear L., Zhong W., Digre A., Katona B., Sjostedt E., Butler L., Odeberg J., Dusart P. (2021). A single-cell type transcriptomics map of human tissues. Sci. Adv..

