# Supplementary material for: Mutant TP53 switches therapeutic vulnerability during gastric cancer progression within interleukin-6 family cytokines
Source: Cell Rep. 2024 Aug 9;43(8):114616. doi: 10.1016/j.celrep.2024.114616 (PMC11372443; doi:10.1016/j.celrep.2024.114616)
Supplement: Document S1. Figures S1–S7 and Tables S1–S5 [file mmc1.pdf]

Cell Reports, Volume *43*

Supplemental information

Mutant TP53 switches therapeutic vulnerability during gastric cancer progression within interleukin-6 family cytokines

Anne Huber, Amr H. Allam, Christine Dijkstra, Stefan Thiem, Jennifer Huynh, Ashleigh R. Poh, Joshua Konecnik, Saumya P. Jacob, Rita Busuttil, Yang Liao, David Chisanga, Wei Shi, Mariah G. Alorro, Stephen Forrow, Daniele V.F. Tauriello, Eduard Batlle, Alex Boussioutas, David S. Williams, Michael Buchert, Matthias Ernst, and Moritz F. Eissmann

A

Pathway	% mutated
Cell cycle	65.8
RTK/RAS	59.5
TP53	56.9
PI3K	41.5
NOTCH	39.2
WNT	37.3
TGF-b	28.7
HIPPO	22.2
MYC	18
NRF2	1.6

B

Pathway	% mutated
RTK/RAS	59.5
PI3K	41.5
TP53	56.9
RTK/RAS + PI3K	26.6
RTK/RAS + TP53	36
PI3K + TP53	24.5
RTK/RAS + PI3K + TP53	15.1

C

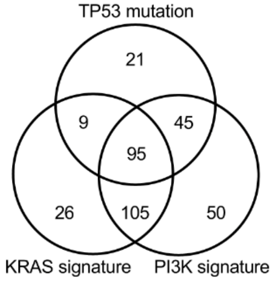

D

Pathway	% activated
KRAS	56.8
PI3K	71.2
TP53*	41
KRAS + PI3K	48.3
KRAS + TP53*	25.1
PI3K + TP53*	33.8
KRAS + PI3K + TP53*	23
none	15.2

* mutation

E

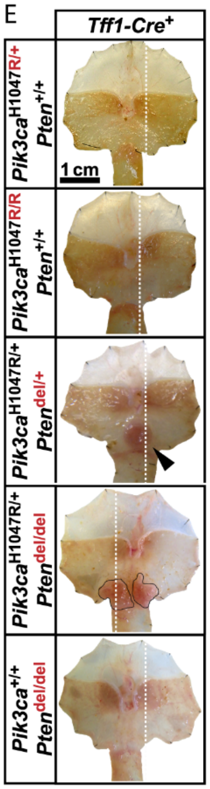

F

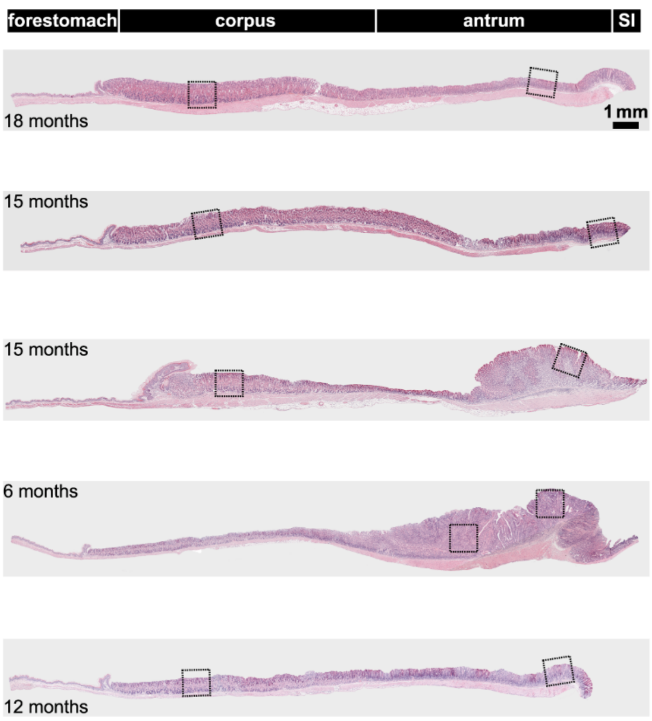

G

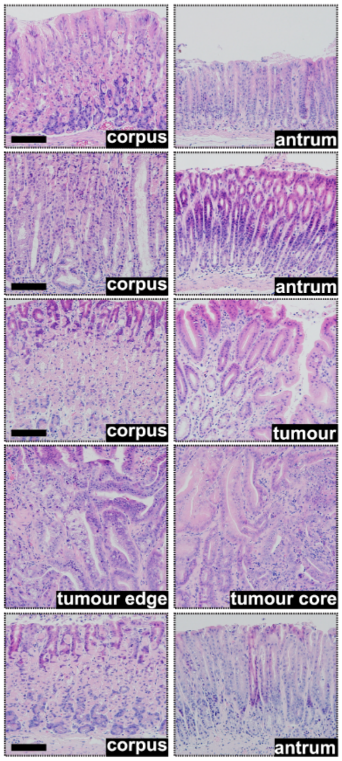

H

Tff1-CreRT2-driven	Tumor (# GC mice/ # mice)	Carcinoma (# Carc mice/ # mice)	Metastasis (# metCarc mice/ # mice)
Pik3ca$^{H1047R/+}$;Pten$^{+/+}$	0% (0/9)	0% (0/9)	0% (0/9)
Pik3ca$^{H1047R/H1047R}$;Pten$^{+/+}$	0% (0/11)	0% (0/11)	0% (0/11)
Pik3ca$^{H1047R/+}$;Pten$^{del/+}$	7.4% (2/27)	0% (0/27)	0% (0/27)
Pik3ca$^{H1047R/+}$;Pten$^{del/del}$	93.8% (15/16)	0% (0/16)	0% (0/16)
Pik3ca$^{+/+}$;Pten$^{del/del}$	0% (0/4)	0% (0/4)	0% (0/4)

I

Tff1-CreRT2-driven	Carcinoma (# Carc mice/ # mice)	p value	Tis (# Tis mice/ # Carc mice)	p value	T1 (# T1 mice/ # Carc mice)	p value	T2 (# T2 mice/ # Carc mice)	p value	T3 (# T3 mice/ # Carc mice)	p value	T4 (# T4 mice/ # Carc mice)	p value
Kras$^{G12D/+}$; Pik3ca$^{H1047R/+}$;Pten$^{del/+}$	56% (9/16)	0.76	22% (2/9)	0.095	56% (5/9)	0.001	11% (1/9)	0.32	11% (1/9)	1	0% 0/9)	0.0001
Kras$^{G12D/+}$; Pik3ca$^{H1047R/+}$;Trp53$^{R172H/+}$	61.3% (19/31)		0% (0/19)		0% (0/19)		0% (0/19)		10.5% (2/19)		89.5% (17/19)	

J

Tff1-CreRT2-driven	Metastasis (# metCarc mice/ # Carc mice)	Liver metastasis only (# Liv Mets mice/ # metCarc mice)	Lung metastasis only (# Lung Mets mice/ # metCarc mice)	Liver+Lung metastasis (# Liv+lung Mets mice/ # metCarc mice)
Kras$^{G12D/+}$;Pik3ca$^{H1047R/+}$;Trp53$^{R172H/+}$	31.6% (6/19)	66.7% (4/6)	0% (0/6)	33.3% (2/6)

Figure S1. Frequency of KRAS, PI3K and TP53 pathway alterations in human gastric cancer and PI3k pathways potency as cancer driver in mice, Related to Figure 1

A Top 10 mutated (in percent) oncogenic pathways in stomach adenocarcinoma (STAD) patients. Data extracted from TCGA data set (STAD n=383). RTK/RAS= Receptor tyrosine kinase/RAS.

B Table shows selected pathways mutation frequencies (as in **A**) for single, double and triple pathway involvement.

C Venn diagram for oncogenic signaling pathway activation gene signature analysis, showing number of patients with STAD with KRAS and/or PI3K pathway activation (based on gene expression signature) and *TP53* gene mutation (silent mutation (n=1) excluded). 63 patients had neither *TP53* mutation nor KRAS or PI3K activation status tumors (STAD n=414).

D Data from **C** presented in table format, showing the precent frequency of patients with single, double, and triple pathways activation (*TP53 data is gene mutation based).

E Representative whole-mount stomachs from Cre-positive mice of the indicated genotypes, collected at 6-18 months (as indicated) after Tamoxifen administration (*arrowhead* and *black lines* indicate tumors).

F,G H&E-stained cross-sections, cut along the dotted line (in **D**), extend from the forestomach to the proximal end of the small intestine (SI) (**E**). Boxed regions are shown in higher magnifications (**F**). Scale bars 100µm.

H Frequency (in percent) of tumor development in Cre-positive mice of the indicated genotype.

I Table shows invasive stages (T stage) of *Kras*$^{LSL\text{-}G12D/+}$*; Pik3ca*$^{LSL\text{-}H1047R/+}$;*Pten*$^{flox/+}$ and *Kras*$^{LSL\text{-}G12D/+}$*; Pik3ca*$^{LSL\text{-}H1047R/+}$;*Trp53*$^{LSL\text{-}R172H/+}$ (*KPT*) carcinoma bearing mice. Tis = carcinoma in situ. P values of Fisher's exact test are shown.

J Table shows frequency of *Kras*$^{LSL\text{-}G12D/+}$*; Pik3ca*$^{LSL\text{-}H1047R/+}$;*Trp53*$^{LSL\text{-}R172H/+}$ (*KPT*) mutant mice bearing metastasis and frequency of their anatomical location.

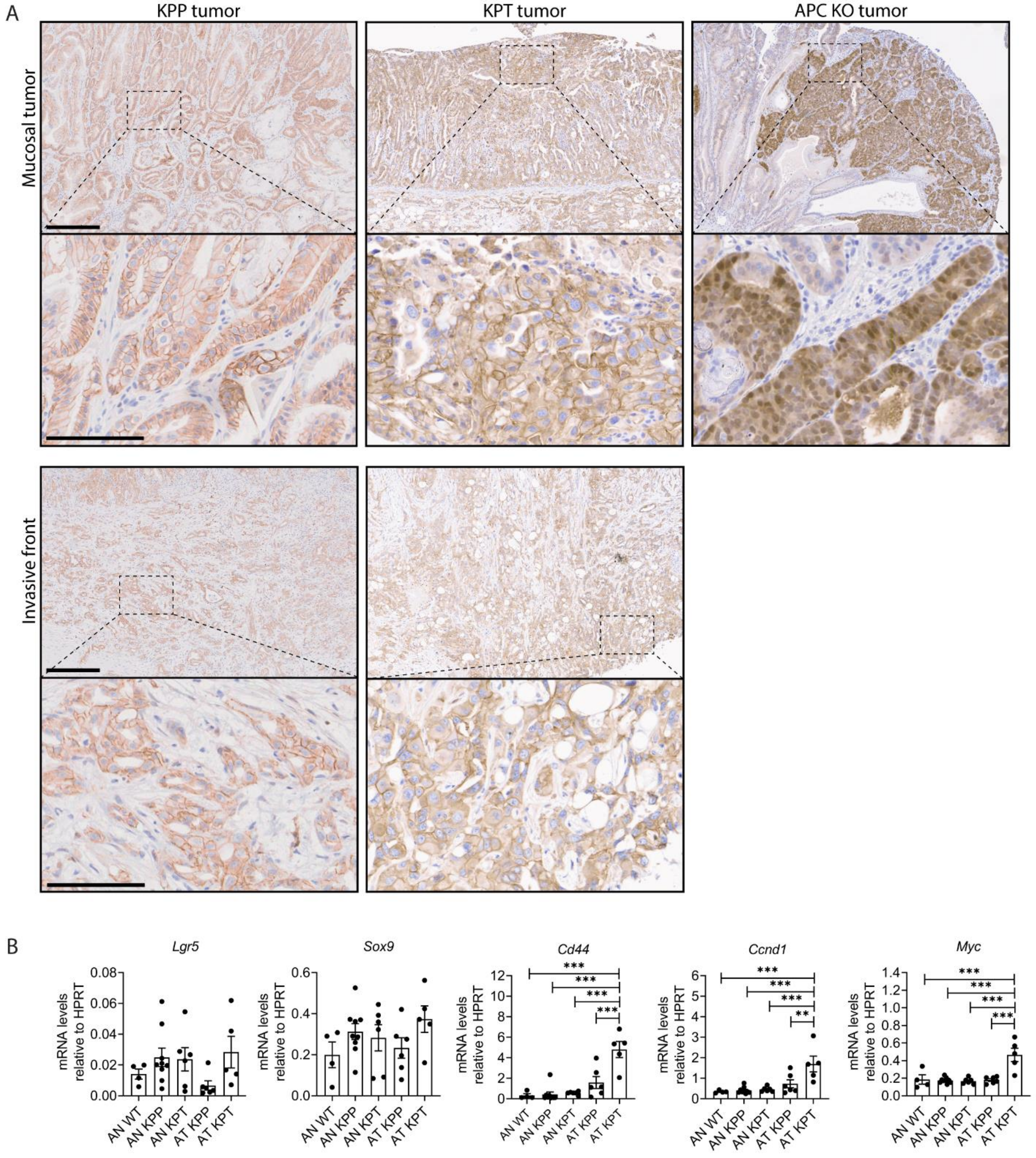

Figure S2. Nuclear β-Catenin and WNT-gene signature analysis in tumors without overt canonical WNT signaling pathway mutations

A Representative microscopy images of anti-β–catenin IHC stained tumors of *KPP*, *KPT* and *Apc*^{KO} (*Tff1*^{CreERT2};*Apc*$^{fl/fl}$) mice. *KPP* and *KPT* image represent strongest staining (of n=10 mice analyzed). Apc^{KO} shown as positive control for nuclear β–catenin positivity; *Apc*^{KO} mice develop non-invasive adenomas. Scale bars equal 300 µm (top images) and 100 µm (bottom images).

B qPCR analysis of transcription levels of canonical WNT signaling target genes of whole tumor tissues. Stomach antrum normal (AN) and antrum tumors (AT) of indicates mutant mice were analyzed. Mean±SEM. One-way ANOVA with Tukey's multiple comparisons test was performed; ns=non-significant, * = p<0.01, ** = p<0.001, *** = p<0.0001. Each data points represents one mouse; N = 4, 9, 6, 6 and 5 (for each graph).

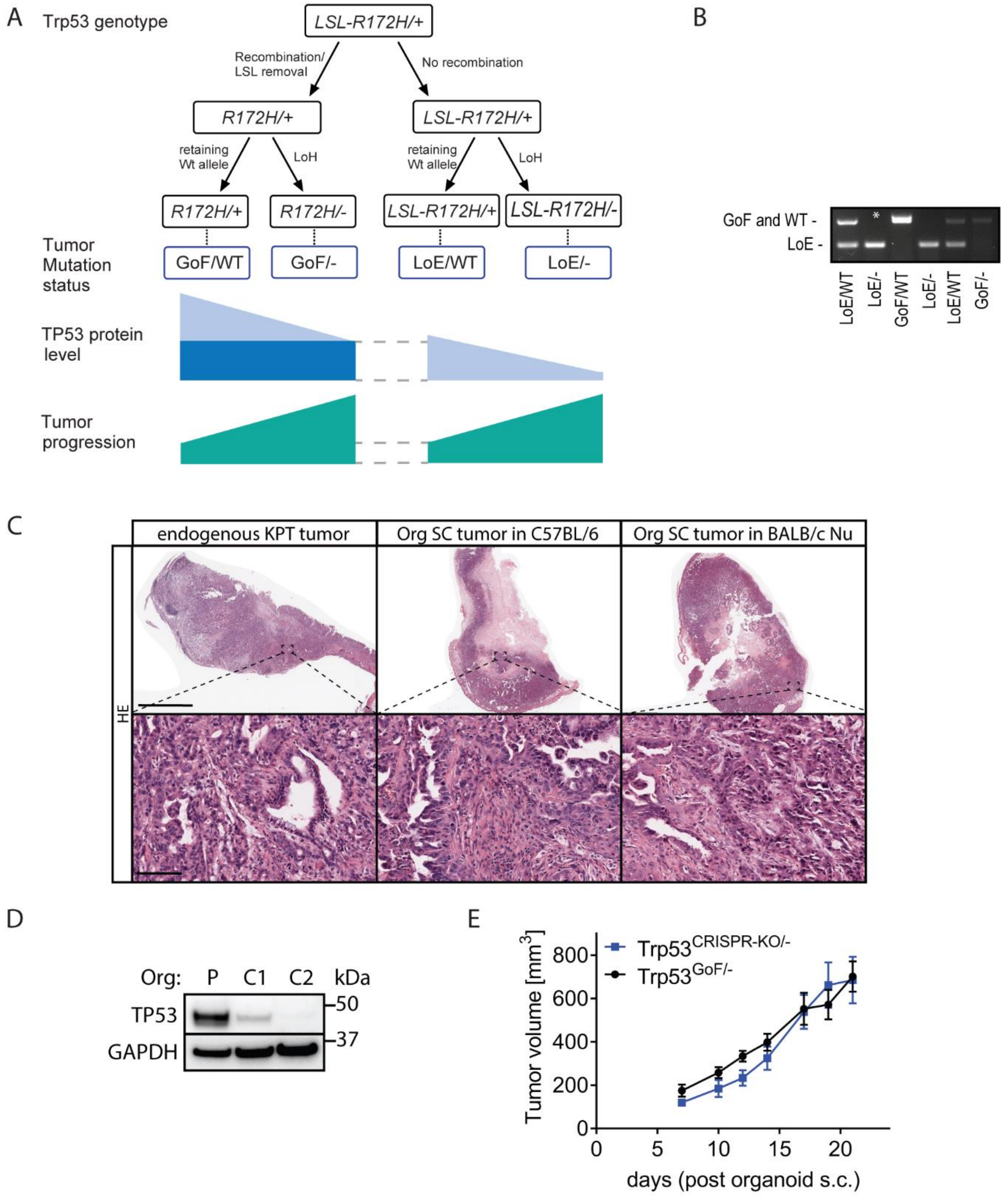

Figure S3. TP53 mutations status at genetic, protein and phenotype level, Related to Figure 2

A Upon tamoxifen administration Tff1^{CreERT2} recombinase drives removal of the Los-Stop-Lox (LSL) cassette from the LSL-R172H allele to yield the R172H mutant expression, which is classified a Gain-of-Function (GoF) mutant. Some cells do not undergo Cre-recombination and retain the unrecombined LSL-R172H allele, which is a Loss-of-Expression (LoE) mutant (or “null’ allele) as no or minimal TP53 protein

can be expressed from this allele. Throughout tumorigenesis and/or tumor progression the wildtype (WT) Trp53 allele can be genetically lost (-), also called Loss-of-heterozygosity. Depending on the mutation status the contribution to expressed protein levels changes between GoF-mutant (dark blue) and WT (light blue) TP53 protein.

B Image showing the secondary *Trp53* status PCR assay performed on genomic DNA of whole tumor or organoid lysates from *KPT* mice. The upper band indicates the presence of the GoF and/or WT allele and the lower band of the LoE allele. The LoE/- tumor organoid sample from Figure 2A is indicated with an asterisk (*).

C Representative H&E images from gastric carcinomas from KPT mice (left), and KPT GC organoid allograft tumors in C57BL/6 (middle) and BALB/C Nude host mice (right). Scalebars = 3mm (top) and 100µm (bottom).

D Immunoblotting for TP53 protein in parental $Trp53^{GoF/-}$ organoids and coisogenic CRISPR-Cas9-mediated $Trp53^{CRISPR\text{-}KO/-}$ daughter organoid clones 1 and 2. $Trp53^{CRISPR\text{-}KO/-}$ clone 2 was selected for further *in vitro* and *in vivo* assays.

E Tumor growth kinetics displayed as calliper measured tumor volumes of subcutaneous transplanted $Trp53^{GoF/-}$ and $Trp53^{CRISPR\text{-}KO/-}$ organoids in C57BL/6 host mice (N=9 and 9) (corresponds to Figure 2H).

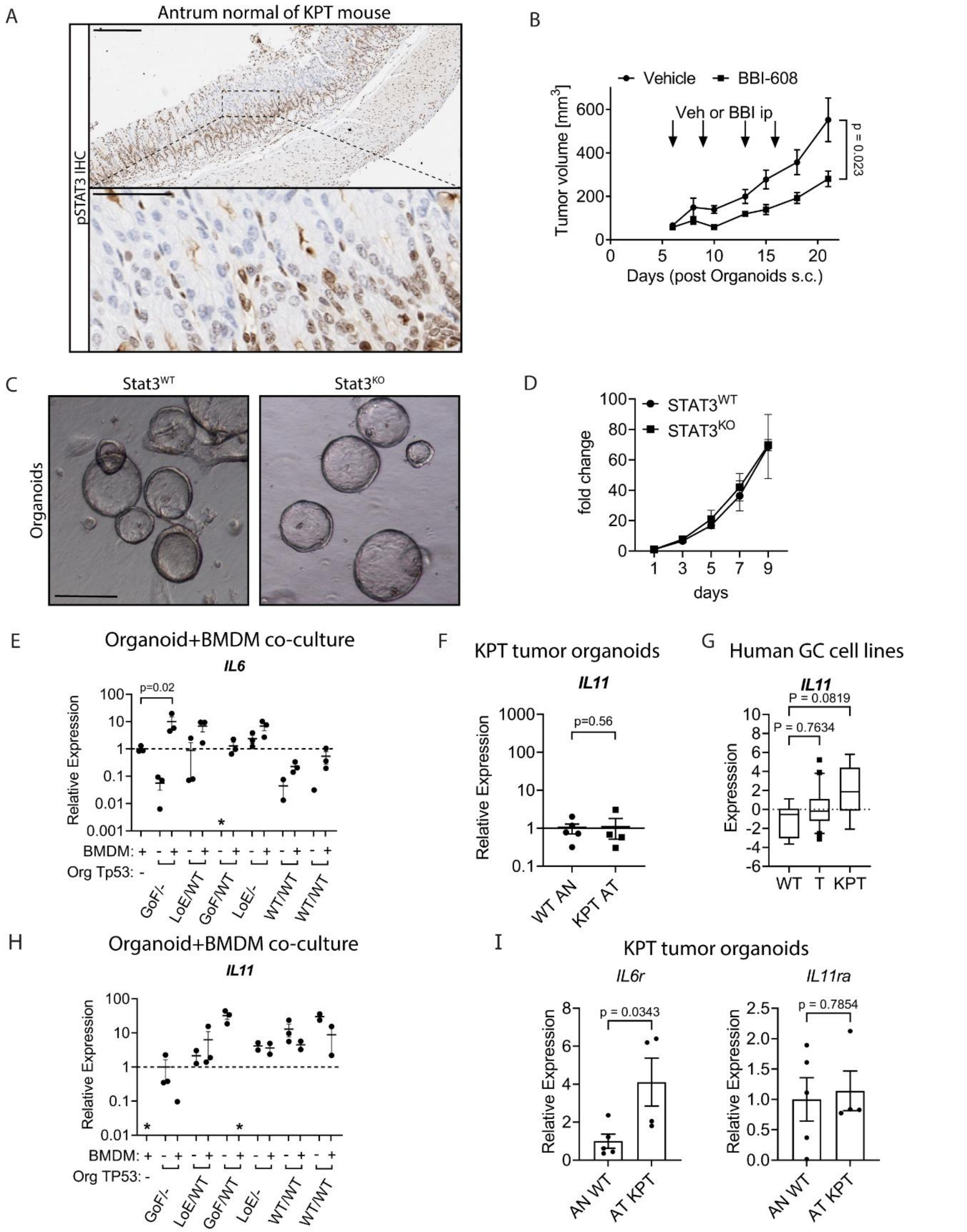

Figure S4. ***IL6*** **and** ***IL11*** **expression profiles in mouse** ***KPT*** **tumor organoids and human GC cells, Related to Figure 3**

A Representative pSTAT3 IHC images of unaffected antrum normal of a KPT mouse showing mucosal and submucosal layers (upper image, scale bar = 200µm) and higher magnification of mucosal compartment (lower image, scale bar = 50µm).

B Tumor volume over time measurements of GC tumor allografts either treated with vehicle or BBI608. (corresponds to Figure 3B, C).

C Representative microscopy images of *Stat3*^{WT} and *Stat3*^{KO} GC organoids (scale bar = 200µm).

D CellTiter-Glo based *in vitro* organoid growth comparison between STAT3^{WT} and STAT3^{KO} coisogenic organoids. N= 3, Mean±SEM.

E qPCR analysis for *IL6* expression of either bone marrow derived macrophages (BMDM) alone, tumor organoids alone (with indicated *TP53* status) or BMDM + organoids co-cultured. The first four organoid cultures are derived from tumors of *KPT* mice and the two *Trp53*$^{WT/WT}$ organoids are derived from *gp130*$^{Y757F/Y757F}$ mouse tumors. Each data point presents the mean of technically triplicates from three independent experiments (some *Trp53*$^{WT/WT}$ organoids alone samples had *IL6* expression below detection limit). Relative expression normalized to BMDM alone. Mean±SEM. One-way ANOVA with Dunnett's multiple comparisons test calculated p value shown, all other comparisons resulted in p>0.2.

F qPCR-determined expression levels of *IL11* in organoids derived from wild type antrum stomach (WT) and stomach tumors of *KPT* mutant mice. Each data points represents organoid from different mice. Expression values were normalized to *Gapdh*. P-values of Mann Whitney test are shown. N = 4,4.

G *IL11* expression levels in human gastric cancer cell lines grouped into *TP53*^{wt} (*WT*), *TP53* (*T*) mutant or *KRAS;PI3K;TP53* (*KPT*) mutant activation signature positive. RNA sequencing data was downloaded from the Broad Institutes Cancer Cell Line Encyclopedia. Data is shown as Box & Whiskers plots (10-90 percentile), comprising WT n=8, T n=21 and KPT n=7 data points. Kruskal-Wallis test and Dunn's multiple comparison's test were performed.

H *IL11* expression analysis from BMDM + organoid co-culture experiment as in **B**. Asterisk (*) indicates sample groups were raw values of each technical replicates from three independent experiments were below the detection limit! Relative expression normalized to GoF/- organoid alone, as *IL11* in BMDM alone expression was below detection limit. Data presentation and statistical testing as in **B**.

I qPCR analysis for expression of *IL6r* (left) and *IL11ra* (right) in organoids derived from wild type antrum stomach (WT) and stomach tumors of *KPT* mutant mice. Each data points represents organoid derived from different mice and expression values are relative to *Gapdh*. P-values of unpaired t-test are shown. N = 5 and 4.

A

Tff1-CreRT2-driven	Carcinoma (# Carc mice/ # mice)	p value	Tis (# Tis mice/ # Carc mice)	p value	T1 (# T1 mice/ # Carc mice)	p value	T2 (# T2 mice/ # Carc mice)	p value	T3 (# T3 mice/ # Carc mice)	p value	T4 (# T4 mice/ # Carc mice)	p value
Kras$^{G12D/+}$; Pik3ca$^{H1047R/+}$;Trp53$^{R172H/+}$	61.3% (19/31)	0.77	0% (0/19)		0% (0/19)		0% (0/19)		10.5% (2/19)	0.63	89.5% (17/19)	0.63
Kras$^{G12D/+}$; Pik3ca$^{H1047R/+}$;Trp53$^{R172H/+}$;Il11ra$^{+/-}$	66.7% (12/18)		0% (0/12)		0% (0/12)		0% (0/12)		16.7% (2/12)		83.3% (10/12)	

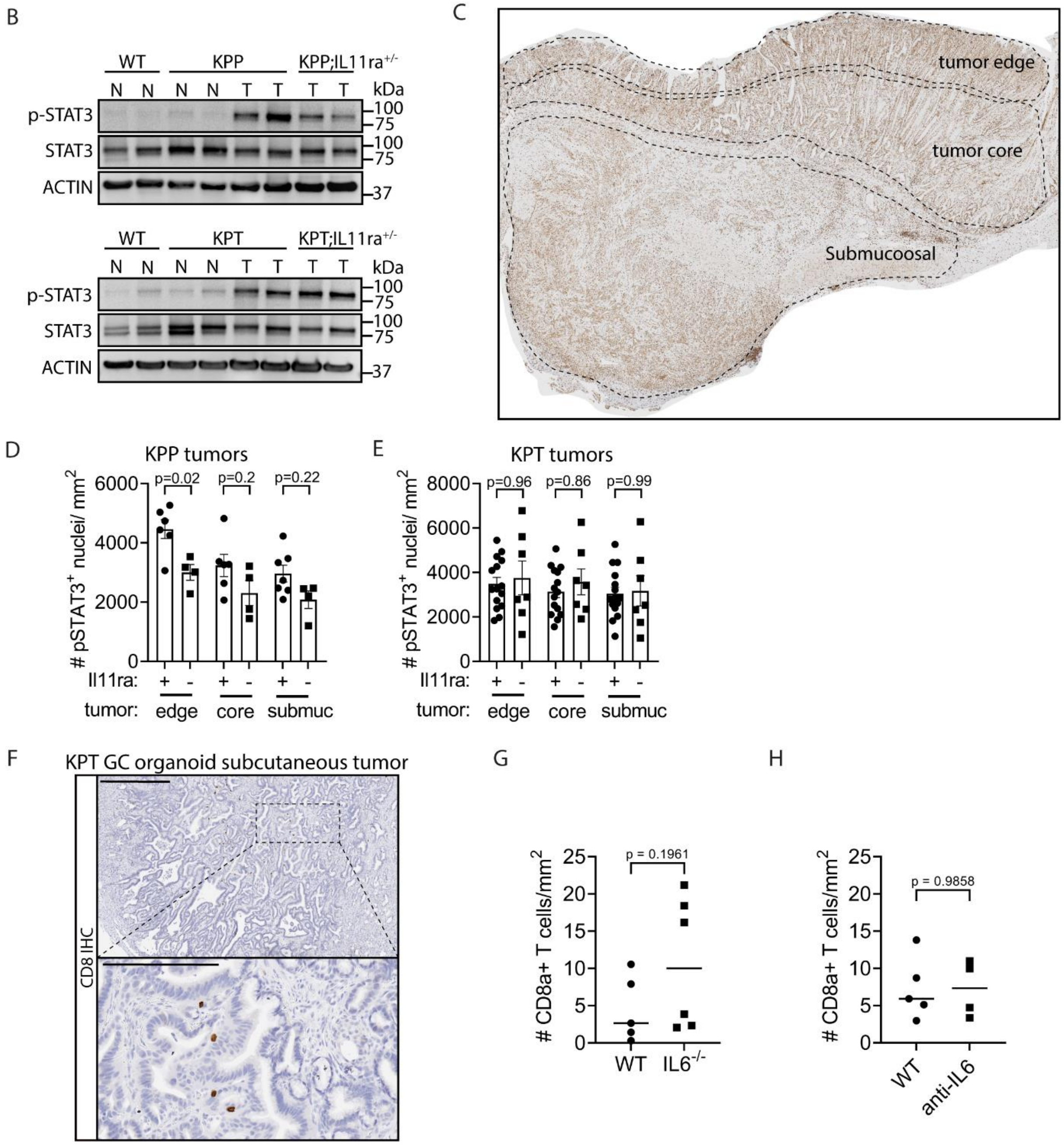

Figure S5. Mutant *Trp53* renders Stat3 signaling activity IL11 independent in *KPT* mutant tumors, Related to Figure 4

A T stage table illustrates depth of invasion for carcinomas with *Kras*$^{LSL-G12D/+}$*; Pik3ca*$^{LSL-H1047R/+}$;*Trp53*$^{LSL-R172H/+}$ (*KPT*) versus *Kras*$^{LSL-G12D/+}$*; Pik3ca*$^{LSL-H1047R/+}$;*Trp53*$^{LSL-R172H/+}$;*Il11ra*$^{+/-}$ genotypes. Tis = carcinoma in situ. P values of Fisher's exact test are shown.

B Immunoblotting for pSTAT3 (pY757), total-STAT3 and ACTIN from tissue lysates from antrum normal (*N*) or tumor (*T*) of wild type (*WT*), *KPP* and *KPP;IL11ra*$^{+/-}$ mice (upper plots) and wild type (*WT*), *KPT* and *KPT;IL11ra*$^{+/-}$ mice (lower plots).

C Representative image of pSTAT3 immunohistochemical stained *KPP* tumor (same as in Fig. 3A) exemplifying the definition of tumor edge, tumor core and submucosal stomach layers containing invaded tumor cells.

D, E Quantification of immunohistochemical staining for pStat3 in FFPE-sections of *KPP* tumors (C) and *KPT* tumors (D). Analyzed tumors were either IL11ra$^{+/+}$ (+) or IL11ra$^{+/-}$ (-). Quantification was performed in tumor edge, tumor core and tumor invaded submucosal layers (as outlined in **B**). P values of one-way ANOVA with Sidak's multiple comparison test are shown. Each data point represents an individual mouse. N = 6, 4 (**D**) and 15, 7 (**E**) (for each edge, core and submuc).

F Representative image of CD8 IHC stained *KPT* GC organoids allograft tumor. Scale bars are 600µm (top) and 200µm (bottom).

G, H Quantification of CD8 positive cells in *KPT* GC organoids allograft tumors grown in WT or IL6$^{-/-}$ mice (**G)**, or treated with isotype control antibody (Ctr) or with anti-IL6 neutralizing antibody (anti-IL6) (**H**) Horizontal line represents mean. Unpaired two-sided T-test P values are shown.

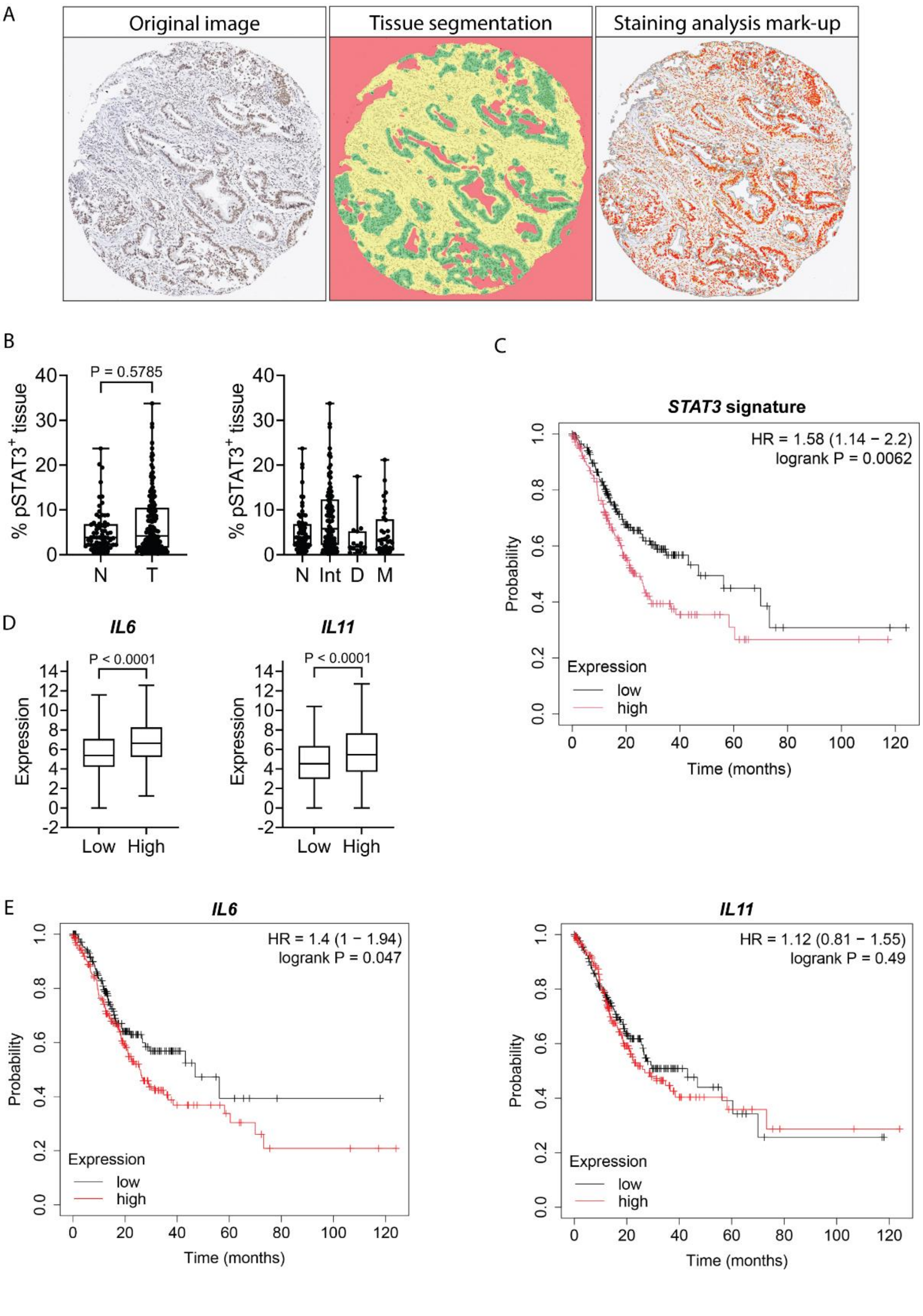

Figure S6. STAT3 signaling quantification and *IL6* cytokine expression on human gastric cancer Tissue Micro Arrays, Related to Figure 5

A Halo analysis of pSTAT3 stained human GC Tumor Micro array (TMA). Left image is the original pSTAT3 image of one representative tumor core of the TMA. Middle image shows the tissue segmentation: halo

AI classified glass/no tissue (red), tumor stroma (yellow) and tumor (green). Right image presents Halo staining quantification Make-up, with weak (yellow), intermediate (orange) and strong (red) pSTAT3 positivity.

B Halo AI staining quantification of pSTAT3 on total tumor core of human GC TMAs (without Halo tissue segmentation). Left graph Box plots: Min to Max showing all data points. N= normal stomach, T= Tumor, Int=Intestinal GC, D=diffuse GC, M=Mixed GC. left: Mann-Whitney, right: Kruskali-Wallis+Dunnet's multiple Comparisons test (all $p>0.11$). N = 68, 168 (left) and 68, 102, 15 and 34 (right).

C Kaplan-Meier survival analysis (overall survival) of STAT3 signaling activation gene signature (STAT3, SOCS3, OSMR, CLDN12, PIM3) stratified by median expression into high versus low in stomach adenocarcinoma (prepared with KM plot). N = 186 (low) and 185 (high).

D mRNA Expression of IL6 and IL11 in Stat3 activation gene signature negative versus positive stomach adenocarcinoma samples (from TCGA data set). Boxplot with error bars showing min to max, p values shown were calculated with Mann-Whitney test. N = 280, 167 (both graphs).

E Kaplan-Meier Survival analysis (overall survival) of IL6 and IL11 high versus low RNA expression in stomach adenocarcinomas (prepared with KMplot). N = 186, 185 (left) and 187, 184 (right); stratified by median gene expression.

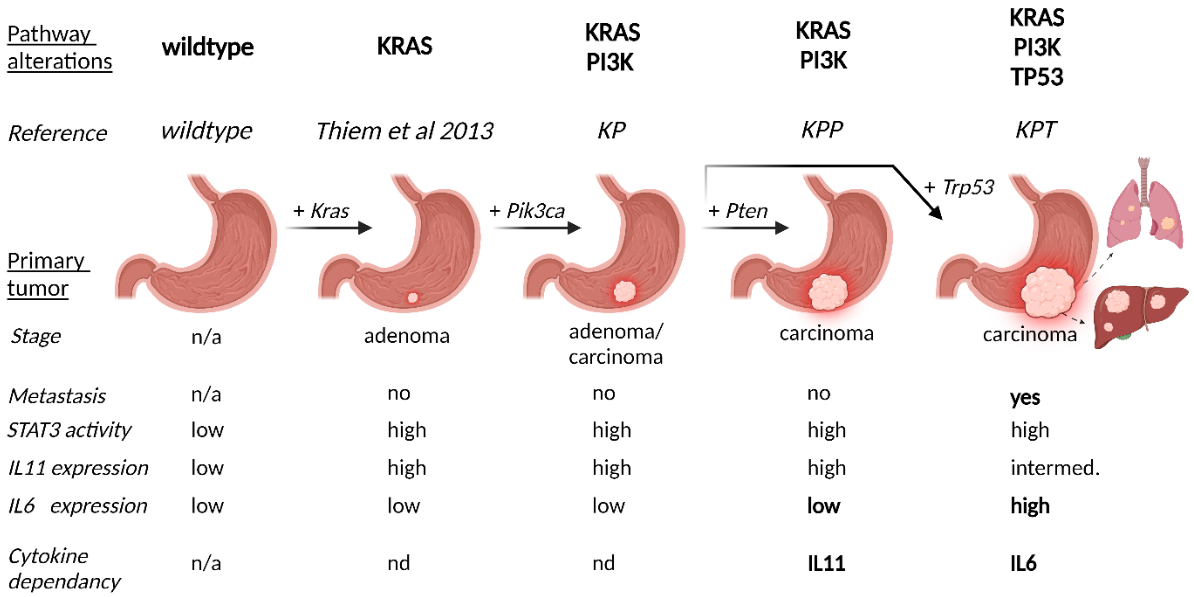

Figure S7. Graphical summary of TP53 mutation driven IL-6 family cytokine dependency switch

Illustrates the pathway activation series with corresponding gastric tumor progression, associated *IL11*, *IL6* expression, STAT3 signalling activity and resulting cytokine dependencies. n/a = not applicable, nd = not determined, intermed. = intermediate. This figure was created with Biorender.com.

Table S1. Histopathological assessment of stomach HE's of tumor-bearing KPT mice, Related to Figure 1 and Figure S1

Diagnosis	WHO subtype	Lauren's subtype	Differentiation grade	Invasion (on slide)	Additional observations
**STAD**	tubular	intestinal	poor	serosa	
**STAD**	tubular	intestinal	moderate/poor	serosa	
**STAD**	mixed	intestinal	poor	subserosa	
**STAD**	solid	intestinal	poor	serosa	spans GOJ
**STAD**	solid	intestinal	poor	serosa	lymph node metastases
**STAD**	mixed	intestinal	moderate/poor	invades liver directly	spans GOJ
**STAD**	solid	intestinal	poor	serosa	
**STAD**	solid	intestinal	poor	serosa	
**STAD**	tubular	intestinal	poor	invades liver directly	lymph node metastasis
**STAD**	tubular	intestinal	moderate	subserosa	lymph node metastasis
**STAD**	tubular	intestinal	poor	into muscularis propria	
**STAD**	tubular	intestinal	poor	serosa	
**STAD**	tubular	intestinal	poor	subserosa	
**STAD**	tubular	intestinal	poor	invades pancreas directly	
**STAD**	tubular	intestinal	moderate	subserosa	lymph node metastasis
**STAD**	tubular	intestinal	poor	invades pancreas directly	
**STAD**	tubular	intestinal	poor	serosa	
**STAD**	tubular	intestinal	moderate	serosa	
**STAD**	tubular	intestinal	poor	invades pancreas directly	

STAD = stomach adenocarcinoma, mixed = mixed tubular and solid WHO subtype, GOJ = Gastric-oesophageal junction

Table S2. Clinicopathological characteristics of GC patient stratified by tumor-compartment pSTAT3 IHC staining, Related to Figure 5 and Figure S6

Characteristic	$pSTAT3^{Low}$ N= 84	$pSTAT3^{High}$ N= 84	p value
**Age (years)**			
Mean ± SD	68.31 ± 10.67	70.31 ± 11.91	0.1
Median	70	72	
Range	44 - 92	27 – 87	
**Site**			
Gastric	68 (81)	63 (75)	0.46
GOJ	16 (20)	21 (25)	
**Lauren subtype**			
Intestinal	43 (57)	59 (79)	0.013
Diffuse	11 (15)	4 (5)	
Mixed	22 (29)	12 (16)	
**AJCC TNM stage**			
1	9 (11)	13 (16)	0.13
2	24 (30)	34 (42)	
3	40 (51)	26 (32)	
4	6 (8)	8 (10)	
**Grade**			
Low	17 (20)	30 (36)	0.03
High	67 (80)	53 (64)	
**Lymph node involvement**			
No	24 (29)	36 (43)	0.08
Yes	58 (71)	47 (57)	
**T4 stage**			
No	50 (61)	61 (73)	0.1
Yes	32 (39)	22 (27)	
**DNA Mismatch Repair status**			
Proficient	66 (80)	69 (82)	0.7
Deficient	17 (20)	15 (18)	

P values are shown from Mann Whitney (Age), Chi-square (AJCC stage, Lauren subtype), Fisher exact (all remaining). Column percentages are shown in brackets. GOJ = Gastric Oesophageal Junction, AJCC= American Joint Committee on Cancer.

Table S3. Tumor, carcinoma, and metastasis incidence of KP, KPP and KPT male and female mice, related to Figure 1 and STAR METHODS

Tff1-CreRT2-driven	Sex	# mice	Tumor (# GC mice/ # mice)	p value	Carcinoma (# Carc mice/ # GC mice)	p value	Metastasis (# metCarc mice/ # Carc mice)	p value
KP	female	12	17% (2/12)	1	50% (1/2)	1	0% (0/1)	1
	male	9	22% (2/9)		50% (1/2)		0% (0/1)	
KPP	female	8	63% (5/8)	0.2	60% (3/5)	1	0% (0/3)	1
	male	8	100% (8/8)		62.5% (5/8)		0% (0/5)	
KPT	female	19	74% (14/19)	0.13	100% (14/14)	1	36% (5/14)	1
	male	12	42% (5/12)		100% (5/5)		20% (1/5)	
KPP;Il11r$^{+/-}$	female	7	29% (2/7)	1	0% (0/2)	1	0% (0/0)	1
	male	6	33% (2/6)		0% (0/2)		0% (0/0)	
KPT;Il11r$^{+/-}$	female	11	73% (8/11)	0.63	100% (8/8)	1	38% (3/8)	1
	male	7	57% (4/7)		100% (4/4)		25% (1/4)	

Table S4. Oligonucleotide sequences for quantitative RT-PCR, Related to STAR METHODS

Gene	Forward Primer	Reverse Primer
Gapdh	CAC TGA GCA TCT CCC TCA CA	GTG GGT GCA GCG AAC TTT AT
Il6	TCTATACCACTTCACAAGTCGGA	GAATTGCCATTGCACAACTCTTT
Il11	ACT TCC ACC TCA GGA CAT CG	GGT GGT TAA AAG GAC AGG CA
Il6r	CCA CGA GT CAG CAC ACA AG	CTA GAG CGG ACA AGC AGA GG
IL11ra	TGG AAG TCC ACC TGA GGA ATG TGT	AGA CCG CAG ACA CTC TCC AAT CAT
Lgr5	CCT TGG CCC TGA ACA AAA TA	ATT TCT TTC CCA GGG AGT GG
Sox9	CCA CGG AAC AGA CTC ACA TCT CTC	CTG CTC AGT TCA CCG ATG TCC ACG
Cd44	GTC TGC ATC GCG GTC AAT AG	GGT CTC TGA TGG TTC CTT GTT
Ccnd1	TCG TGG CCT CTA AGA TGA AGG A	TCG GGC CGG ATA GAG TTG T
Myc	AGC TGT TTG AAG GCT GGA TT	AAT AGG GCT GTA CGG AGT CG
Hprt	GGC CAG ACT TTG TTG GAT TTG	CGC TCA TCT TAG GCT TTG TAT TTG

Table S5. Primer sequences used for PCR A and B for Trp53 status analysis, Related to STAR METHODS

Primer Name	Primer Sequence
**PCR A**	
Olive2004_LSLcas-Recomb_F	agc ctg cct agc ttc ctc agg
Olive2004_LSLcas-Recomb_R	ctt gga gac ata gcc aca ctg
**PCR B**	
Trp53 WT	agg tgt ggc ttc tgg ctt c
Trp53 neo mutant	cca tgg ctt gag taa gtc tgc a
Trp53 common	gaa act ttt cac aag aac cag atc a
